# Supplementary material for: Secretin targets interstitial cells of Cajal to regulate intestinal contractions
Source: EMBO Rep. 2025 Nov 6;26(23):6015–43. doi: 10.1038/s44319-025-00623-1 (PMC12678811; doi:10.1038/s44319-025-00623-1)
Supplement: Supplementary file 1 — Appendix [file 44319_2025_623_MOESM1_ESM.pdf]

TABLE OF CONTENTS

1 **Appendix Figures**

1.1 Appendix Figure S1 1
low concentration of secretin effects on murine small intestinal muscle contractions.
1.2 Appendix Figure S2 2
Normal contractile activity returns after secretin washout in small intestinal muscle strips.
1.3 Appendix Figure S3 3
RNAScope control probes on jejunal tissue.

2 **Appendix Tables**

2.1 Appendix Table S1 4
Summary analysis of murine muscle contraction parameters in response to secretin and TTX.
2.2 Appendix Table S2 5
Summary analysis of macaca fascicularis muscle contraction parameters in response to secretin and TTX.
2.3 Appendix Table S3 6
Summary analysis of Ca^{2+} transient parameters of ICC-DMP and ICC-MY in response to secretin and TTX.
2.4 Appendix Table S4 7
Summary analysis of Ca^{2+} transient parameters of ICC-DMP in response to EFS.
2.5 Appendix Table S5 8
Summary analysis of Ca^{2+} transient parameters of ICC-DMP in response to IP3 uncaging.
2.6 Appendix Table S6 9
Summary analysis of Ca^{2+} transient parameters of ICC-DMP in response to nominal external Ca^{2+}solution.
2.7 Appendix Table S7 10
Summary analysis of Gs-DREADD-Kit muscle contraction parameters before and after CNO.
2.8 Appendix Table S8 11
Summary analysis of cAMP analogue and activator on murine contraction parameters.
2.9 Appendix Table S9 12
Summary analysis of Ca^{2+} transient parameters of ICC-DMP and ICC-MY in response to 8-bromo-cAMP.
2.10 Appendix Table S10 13
Summary analysis of FRET of cAMP sensor in ICC-DMP in response to secretin and forskolin.
2.11 Appendix Table S11 14
Summary analysis of murine contraction parameters in response to AT7867 and secretin.
2.12 Appendix Table S12 15
Summary analysis of Ca^{2+} transient parameters of ICC-DMP in the presence of AT7867 and secretin.
2.12 Appendix Table S13 16
Summary analysis of murine contraction parameters in response to ESI-05 and secretin.
2.12 Appendix Table S14 17
Summary analysis of Ca^{2+} transient parameters of ICC-DMP in the presence of ESI-05 and secretin.

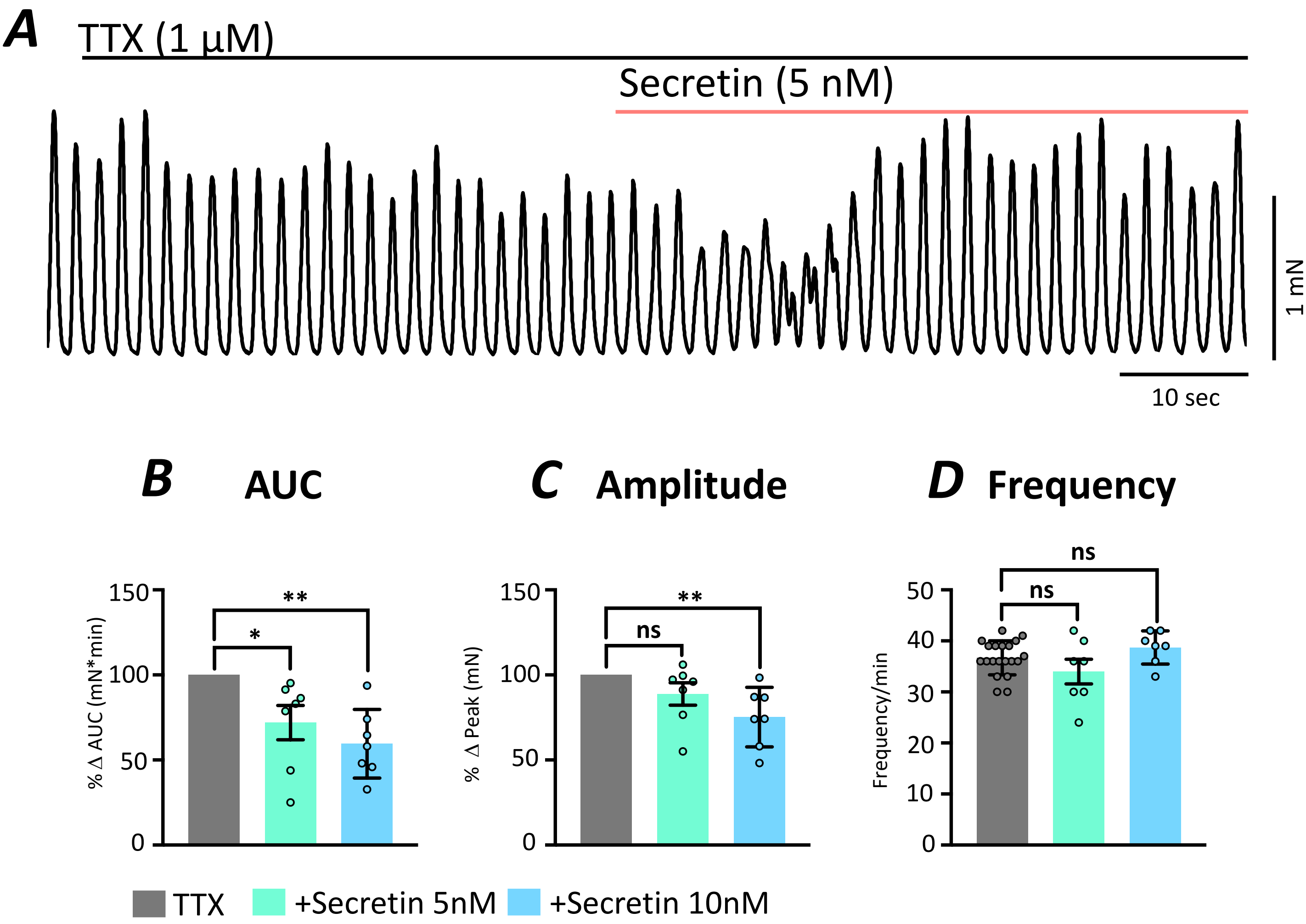

Appendix Figure S1 : Secretin inhibits murine small intestinal muscle contractions. Contractions of jejunal muscle strips in secretin (5 nM and 10 nM) in the presence of tetrodotoxin (TTX; 1 µM). (A) Small intestine contraction trace of secretin 5 nM in the presence of TTX. The contractions were tabulated as area under the curve (AUC), amplitude and frequency. (B) Area under the curve (AUC; mN*min) and (C) amplitude (mN) were reduced by secretin, but (D) the frequency (min-1) of contractions was unaffected. All data were normalized to control responses, except the contraction frequency. The data are plotted as mean ± SEM, and significance was determined using Student's paired t-test, **** = P<0.0001; Strips =7, n=4 for secretin (5 nM) and strips =7, n=5 for secretin (10 nM).

Appendix Figure S1

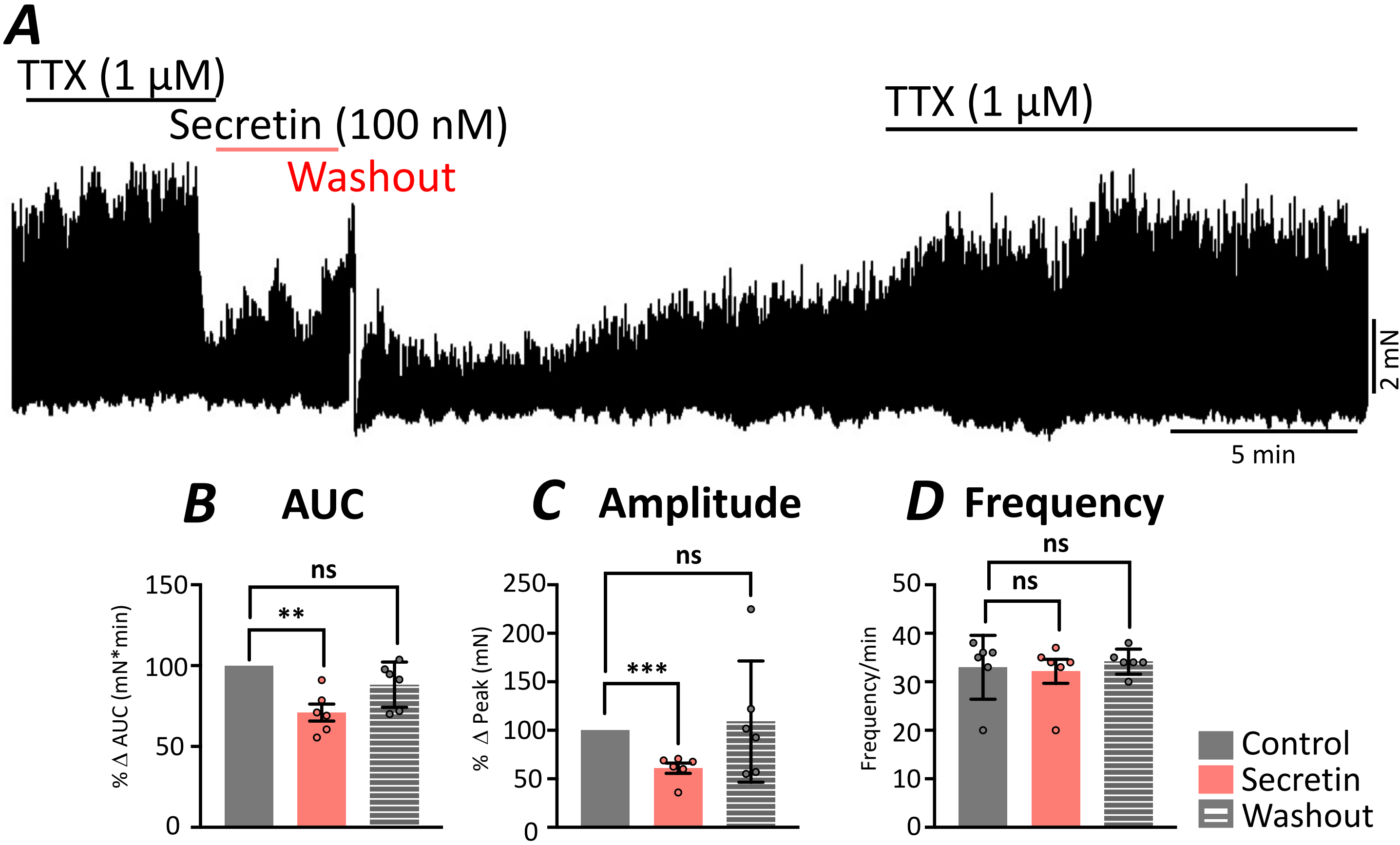

Appendix Figure S2: Normal contractile activity returns after secretin washout in small intestinal muscle strips.
Contractions of jejunal muscle strips in secretin 100 nM in the presence of tetrodotoxin (TTX; 1 µM). (A) Small intestine contraction trace of secretin 100 nM in the presence of TTX and normal activity after a washout. (B) Area under the curve (AUC; mN*min) and (C) amplitude (mN) were reduced by secretin, but (D) the frequency (min-1) of contractions was unaffected. All data were normalized to control responses, except the contraction frequency. The data are plotted as mean ± SEM, and significance was determined using Student's paired t-test, **** = P<0.0001; Strips =6, N=3 for all.

Appendix Figure S2

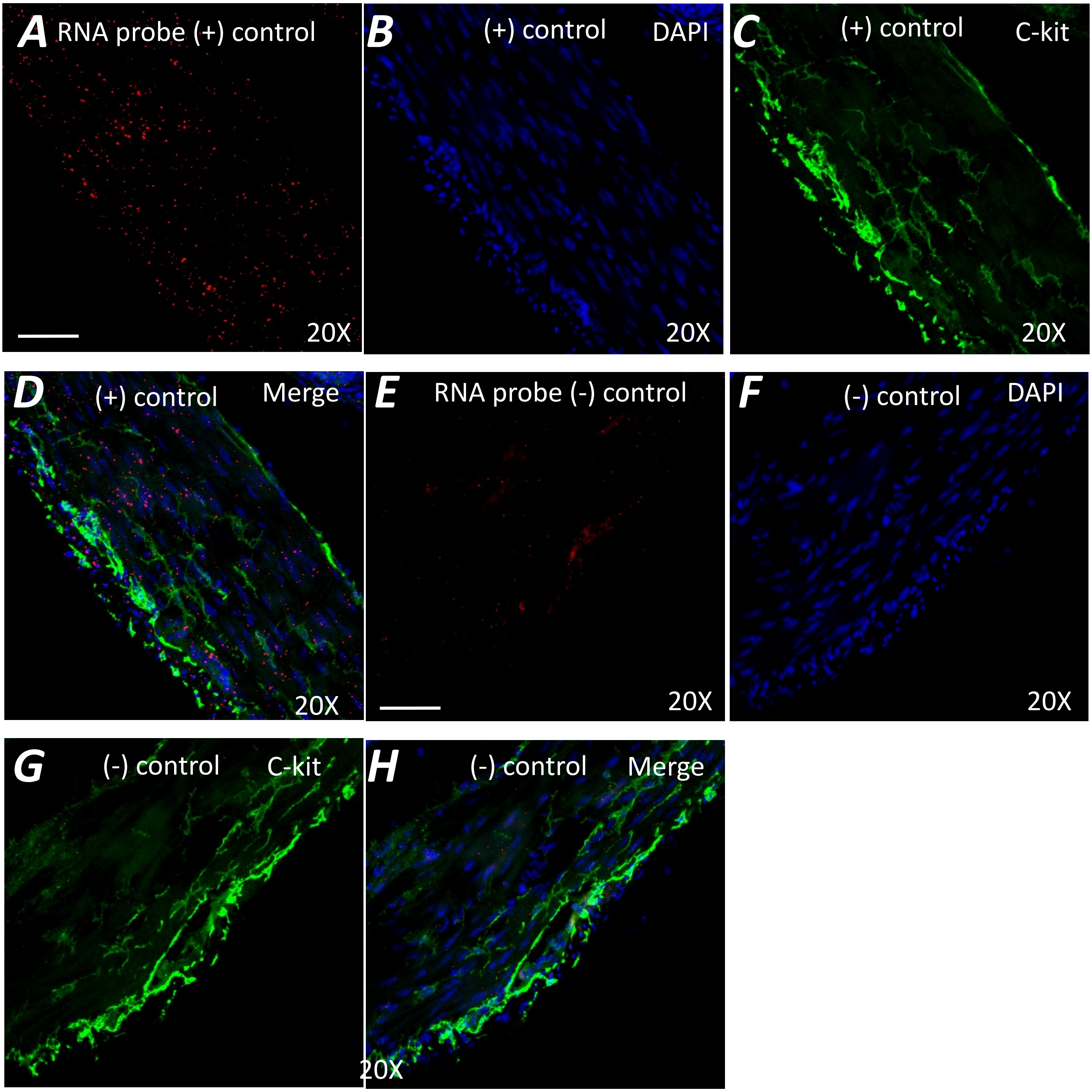

Appendix Figure S3: RNAScope control probes on jejunal tissue.
(A) Confocal image of murine jejunal cross-section at 20x magnification and visualization of the RNAscope probe for positive control probe (red). (B) DAPI nuclear staining of image A. (C) Antibodies immunostaining of c-Kit (green) of ICC. (D) Merge image of A, B and C. (E) visualization of the RNAscope probe for negative control probe (red). (F) DAPI nuclear staining of image E. (G) Antibodies immunostaining of c-Kit (green) of ICC. (H) Merge image of E, F and G.

Appendix Figure S3

Appendix Table S1: **Summary analysis of murine muscle contraction parameters (frequency, AUC and amplitude) in response to secretin and TTX.**

**Contractile parameter** **Murine small intestine**	**Control**	**Secretin (100nM)**		
	Mean-SEM	Mean-SEM	P value	N
Frequency	34 ± 0.5054	33.65 ± 0.5376	0.0589 ns	8
AUC (mN*min)	100%	45.68% ± 3.88	<0.0001 ****	8
Peak amplitude (mN)	100%	58.43% ± 4.559	<0.0001 ****	8

**Contractile parameter** **Murine small intestine**	**TTX (1µM)**	**TTX +Secretin (100nM)**		
	Mean-SEM	Mean-SEM	P value	N
Frequency	36.85 ± 1.25	36.38 ± 1.357	0.3370 ns	7
AUC (mN*min)	100%	46.35% ± 4.507	<0.0001 ****	7
Peak amplitude (mN)	100%	53.28% ± 3.365	<0.0001 ****	7

**Contractile parameter** **Murine small intestine**	**Secretin (100nM)**	**TTX +Secretin (100nM)**		
	Mean-SEM	Mean-SEM	P value	N
Frequency	33.65 ± 0.5376	36.38 ± 1.357	0.9112 ns	8
AUC (mN*min)	45.68% ± 3.88	46.35% ± 4.507	0.3689 ns	8
Peak amplitude (mN)	58.43% ± 4.559	53.28% ± 3.365	0.0798 ns	8

P value < 0.0001 are represented by four asterisks (****). No significance refers to ns. N refers to the number of animals.

Appendix Table S2: **Summary analysis of macaca fascicularis muscle contraction parameters (frequency, AUC and amplitude) in response to secretin and TTX.**

**Contractile parameter** macaca fascicularis small intestine	**Control**	**Secretin (100nM)**		
	Mean-SEM	Mean-SEM	P value	N
Frequency	9.6 ± 1.448	9.64 ± 2.36	0.6797 ns	3
AUC (mN*min)	100%	59.32% ± 8.916	0.0002 ***	3
Peak amplitude (mN)	100%	45.76% ± 9.039	0.0150 *	3

**Contractile parameter** macaca fascicularis small intestine	**TTX (1μM)**	**TTX +Secretin (100nM)**		
	Mean-SEM	Mean-SEM	P value	N
Frequency	8.8 ± 2.066	8.467 ± 2.256	0.2149 ns	4
AUC (mN*min)	100%	59.6%2 ± 7.286	0.0026 **	4
Peak amplitude (mN)	100%	51.0% ± 9.052	0.0029 **	4

P value < 0.05 are represented by a single asterisk (*), P value < 0.01 are represented by two asterisks (**), P value < 0.001 are represented by three asterisks (***). No significance refers to ns. N refers to the number of animals.

Appendix Table S3: **Summary analysis of Ca^{2+} transient parameters (frequency, spatial spread, duration and event area) of ICC-DMP and ICC-MY in response to secretin and TTX.**

**Calcium Signal Parameter ICC-DMP**	**TTX (1µM)**	**TTX +Secretin (100nM)**		
	Mean-SEM	Mean-SEM	P value	N
Frequency (Ca^{2+} transients/30s)	10.52 ± 1.152	3.108 ± 1.109	<0.0001 ****	8
Spatial Spread (µm)	10.52 ± 1.152	3.108 ± 1.109	0.0002 ***	8
Duration (ms)	3.151 ± 0.2191	1.195 ± 0.3939	0.0001 ***	8
Area (Distance µm*s)	2.007 ± 0.2859	0.5708 ± 0.241	0.0001 ***	8

**Calcium Signal Parameter ICC-MY**	**TTX (1µM)**	**TTX +Secretin (100nM)**		
	Mean-SEM	Mean-SEM	P value	N
Frequency (Ca^{2+} transients/30s)	18.85 ± 3.595	18.79 ± 3.717	0.2100 ns	6
Spatial Spread (µm)	18.85 ± 3.595	18.79 ± 3.717	0.9121 ns	6
Duration (ms)	7.95 ± 0.8004	8.284 ± 0.8808	0.3380 ns	6
Area (Distance µm*s)	11 ± 2.117	11.77 ± 2.192	0.1540 ns	6

P value < 0.001 are represented by three asterisks (***). P value < 0.0001 are represented by four asterisks (****) No significance refers to ns. N refers to the number of animals.

Appendix Table S4: **Summary analysis of Ca^{2+} transient parameters (frequency, spatial spread, duration and event area) of ICC-DMP in response to EFS.**

**Calcium Signal Parameter ICC-DMP**	**Control** EFS: (3Hz, continuous)	**Secretin (100nM)** EFS: (3Hz, continuous)		
	Mean-SEM	Mean-SEM	P value	N
Frequency (Ca^{2+} transients/30s)	26.16 ± 2.845	1.4 ± 0.4619	<0.0001 ****	3
Spatial Spread (µm)	11.23 ± 0.5336	4.346 ± 1.211	<0.0001 ****	3
Duration (ms)	2.527 ± 0.06578	0.9946 ± 0.2541	<0.0001 ****	3
Area (Distance µm*s)	1.741 ± 0.09827	0.6907 ± 0.2034	0.0002 ***	3

**Calcium Signal Parameter ICC-DMP**	**Control-Before** EFS (10Hz for 10s)	**Control-Post stimulation** EFS (10Hz for 10s)		
	Mean-SEM	Mean-SEM	P value	N
Frequency (Ca^{2+} transients/30s)	26.4 ± 6.493	41.7 ± 5.053	0.0022 **	5
Spatial Spread (µm)	12.29 ± 1.039	12.71 ± 1.615	0.7219 ns	5
Duration (ms)	3.189 ± 0.1849	3.659 ± 0.5134	0.3574 ns	5
Area (Distance µm*s)	2.408 ± 0.2819	2.572 ± 0.435	0.6207 ns	5

**Calcium Signal Parameter ICC-DMP**	**Secretin-Before** EFS (10Hz for 10s)	**Secretin-Post stimulation** EFS (10Hz for 10s)		
	Mean-SEM	Mean-SEM	P value	N
Frequency (Ca^{2+} transients/30s)	2.091 ± 1.148	3.364 ± 1.485	0.3559 ns	5
Spatial Spread (µm)	4.584 ± 1.537	5.969 ± 2.124	0.7041 ns	5
Duration (ms)	1.298 ± 0.3913	1.256 ± 0.4598	0.9480 ns	5
Area (Distance µm*s)	0.6689 ± 0.26	0.9574 ± 0.3933	0.5837 ns	5

P value < 0.01 are represented by two asterisks (**), P value < 0.001 are represented by three asterisks (***). P value < 0.0001 are represented by four asterisks (****). No significance refers to ns. N refers to the number of animals. EFS: Electrical field stimulation either continuous at 3 Hz or a stimulus at (10Hz, for 10 sec).

Appendix Table S5: **Summary analysis of Ca^{2+} transient parameters (frequency, spatial spread, duration and event area) of ICC-DMP in response to IP3 uncaging.**

**Calcium Signal Parameter ICC-DMP**	**Control Before IP3 uncaging**	**Control After IP3 uncaging**		
	Mean-SEM	Mean-SEM	P value	N
Frequency (Ca^{2+} transients/30s)	19.27 ± 5.702	30.27 ± 5.552	0.0002 ***	4
Spatial Spread (µm)	10.08 ± 1.433	12.32 ± 1.386	0.0861 ns	4
Duration (ms)	2.977 ± 0.1665	4.136 ± 0.6184	0.0420 *	4
Area (Distance µm*s)	1.878 ± 0.2828	3.089 ± 0.6703	0.0621 ns	4

**Calcium Signal Parameter ICC-DMP**	**Secretin Before IP3 uncaging**	**Secretin After IP3 uncaging**		
	Mean-SEM	Mean-SEM	P value	N
Frequency (Ca^{2+} transients/30s)	0.6 ± 0.4	1.273 ± 0.8954	0.2100 ns	4
Spatial Spread (µm)	4.555 ± 2.52	9.225 ± 4.36	0.2685 ns	4
Duration (ms)	1.063 ± 0.6068	1.355 ± 0.5568	0.5398 ns	4
Area (Distance µm*s)	0.8799 ± 0.5496	1.866 ± 0.9586	0.3354 ns	4

P value < 0.05 are represented by three asterisks (*). P value < 0.001 are represented by three asterisks (***). No significance refers to ns. N refers to the number of animals.

Appendix Table S6: **Summary analysis of Ca^{2+} transient parameters (frequency, spatial spread, duration and event area) of ICC-DMP in response to nominal external Ca^{2+}solution.**

**Calcium Signal Parameter ICC-DMP**	**Control 2 mM Ca^{2+}**	**Secretin (100nM) 2 mM Ca^{2+}**		
	Mean-SEM	Mean-SEM	P value	N
Frequency (Ca^{2+} transients/30s)	50.67 ± 16.28	4.667 ± 4.08	0.0445 *	5
Spatial Spread (µm)	8.881 ± 0.4967	4.203 ± 2.262	0.0599 ns	5
Duration (ms)	4.051 ± 0.7989	1.144 ± 0.5522	0.0587 ns	5
Area (Distance µm*s)	2.103 ± 0.3982	0.6425 ± 0.4484	0.0816 ns	5

**Calcium Signal Parameter ICC-DMP**	**Nominal-Ca^{2+}**	**Secretin (100nM) Nominal-Ca^{2+}**		
	Mean-SEM	Mean-SEM	P value	N
Frequency (Ca^{2+} transients/30s)	41.4 ± 9.32	0.60 ± 0.40	0.0111 *	5
Spatial Spread (µm)	10.38 ± 1.569	5.636 ± 3.452	0.1413 ns	5
Duration (ms)	3.574 ± 0.3078	1.334 ± 0.8182	0.0609 ns	5
Area (Distance µm*s)	2.366 ± 0.4062	0.8848 ± 0.5452	0.0145 *	5

P value < 0.05 are represented by three asterisks (*). No significance refers to ns. N refers to the number of animals.

Appendix Table S7: **Summary analysis of Gs-DREADD-Kit muscle contraction parameters (frequency, AUC, amplitude and tone) before and after CNO.**

**Contractile parameter** **Murine small intestine**	**Control** **Gs-DREADD-Kit**	**CNO (10µM)** **Gs-DREADD-Kit**		
	Mean-SEM	Mean-SEM	P value	N
Frequency	31.85 ± 1.846	31.38 ± 1.817	0.5845 ns	5
AUC (mN*min)	100%	56.0% ± 6.756	<0.0001 ****	5
Peak amplitude (mN)	100%	67.31% ± 4.815	<0.0001 ****	5
Tone(mN)	100%	87.15% ± 7.869	0.1285 ns	5

P value < 0.0001 are represented by four asterisks (****). No significance refers to ns. N refers to the number of animals.

Appendix Table S8: **Summary analysis of cAMP analogue and activator on murine contraction parameters (frequency, AUC and amplitude).**

**Contractile parameter** Murine small intestine	**Control**	**Forskolin (20nM)**		
	Mean-SEM	Mean-SEM	P value	N
Frequency	31.17 ± 2.664	33.55 ± 1.617	0.1566 ns	8
AUC (mN*min)	100%	38.33% ± 5.933	<0.0001 ****	8
Peak amplitude (mN)	100%	51.56% ± 5.602	<0.0001 ****	8

**Contractile parameter** Murine small intestine	**Control**	**8-Bromo-cAMP (500µM)**		
	Mean-SEM	Mean-SEM	P value	N
Frequency	35.05 ± 1.313	30.5 ± 2.907	0.3939 ns	4
AUC (mN*min)	100%	21.22% ± 5.239	<0.0001 ****	4
Peak amplitude (mN)	100%	36.7% ± 7.145	0.0003 ***	4

P value < 0.001 are represented by three asterisks (***). P value < 0.0001 are represented by four asterisks (****). No significance refers to ns. N refers to the number of animals.

Appendix Table S9: **Summary analysis of Ca^{2+} transient parameters (frequency, spatial spread, duration and event area) of ICC-DMP and ICC-MY in response to 8-bromo-cAMP.**

**Calcium Signal Parameter ICC-DMP**	**TTX (1μM)**	**TTX + 8-bromo-cAMP (500μM)**		
	Mean-SEM	Mean-SEM	P value	N
Frequency (Ca^{2+} transients/30s)	38.33 ± 8.841	11.33 ± 2.944	0.0032 **	4
Spatial Spread (μm)	9.07 ± 0.9338	6.347 ± 1.136	0.0383 *	4
Duration (ms)	2.888 ± 0.1525	2.569 ± 0.4053	0.5381 ns	4
Area (Distance μm*s)	1.575 ± 0.2153	1.102 ± 0.2079	0.0616 ns	4

**Calcium Signal Parameter ICC-MY**	**TTX (1μM)**	**TTX + 8-bromo-cAMP (500μM)**		
	Mean-SEM	Mean-SEM	P value	N
Frequency (Ca^{2+} transients/30s)	20.9 ± 2.885	20.2 ± 2.52	0.7286 ns	3
Spatial Spread (μm)	11.41 ± 1.249	11.39 ± 2.058	0.9855 ns	3
Duration (ms)	3.747 ± 0.2993	3.669 ± 0.5988	0.8486 ns	3
Area (Distance μm*s)	2.93 ± 0.5755	3.315 ± 1.108	0.5607 ns	3

P value < 0.05 are represented by three asterisks (*). P value < 0.01 are represented by three asterisks (**). No significance refers to ns. N refers to the number of animals.

Appendix Table S10: **Summary analysis of FRET of cAMP sensor in ICC-DMP in response to secretin and forskolin.**

**Imaging FRET parameter**<br>**Murine small intestine**	**Control**<br>**CAMPER-Kit-iCre**	**DMSO**		
	Mean-SEM	Mean-SEM	P value	N
% CFP/YFP	100%	102% ± 1.526	0.268<br>ns	5
**Imaging FRET parameter**<br>**Murine small intestine**	**Control**<br>**CAMPER-Kit-iCre**	**Secretin (100nM)**		
	Mean-SEM	Mean-SEM	P value	N
% CFP/YFP	100%	107.9% ±<br>0.871	<0.0001<br>****	30
**Imaging FRET parameter**<br>**Murine small intestine**	**Control**<br>**CAMPER-Kit-iCre**	**Forskolin (1μM)**		
	Mean-SEM	Mean-SEM	P value	N
% CFP/YFP	100%	115% ± 2.269	<0.0001<br>****	11

P value < 0.0001 are represented by four asterisks (****). No significance refers to ns. N refers to the number of cells.

Appendix Table S11: **Summary analysis of murine contraction parameters (frequency, AUC and amplitude) in response to AT7867 and secretin.**

**Contractile parameter** Murine small intestine	**Control**	**Secretin (100nM)**		
	Mean-SEM	Mean-SEM	P value	N
Frequency	31.64 ± 2.188	31.21 ± 2.418	0.6276 ns	7
AUC (mN*min)	100%	49.59v ± 6.215	<0.0001 ****	7
Peak amplitude (mN)	100%	62.6% ± 6.177	0.0001 ***	7

**Contractile parameter** Murine small intestine	**AT7867 (10µM)**	**AT7867 + Secretin**		
	Mean-SEM	Mean-SEM	P value	N
Frequency	31.2 ± 1.533	30.9 ± 1.449	0.0607 ns	4
AUC (mN*min)	100%	98.48% ± 15.64	0.9247 ns	4
Peak amplitude (mN)	100%	86.64% ± 8.202	0.1378 ns	4

**Contractile parameter** Murine small intestine	**Control**	**AT7867**		
	Mean-SEM	Mean-SEM	P value	N
Frequency	34.3 ± 2.044	31.2 ± 1.533	0.0036 **	4
AUC (mN*min)	100%	120.8% ± 36.83	0.5856 ns	4
Peak amplitude (mN)	100%	105.6% ± 21.73	0.8028 ns	4

P value < 0.05 are represented by three asterisks (*). P value < 0.001 are represented by three asterisks (***). P value < 0.0001 are represented by four asterisks (****). No significance refers to ns. N refers to the number of animals

Appendix Table S12: **Summary analysis of Ca^{2+} transient parameters (frequency, spatial spread, duration and event area) of ICC-DMP in the presence of AT7867 and secretin.**

**Calcium Signal Parameter ICC-DMP**	**Control**	**Secretin (100nM)**		
	Mean-SEM	Mean-SEM	P value	N
Frequency (Ca^{2+} transients/30s)	41.74 ± 3.607	3.118 ± 1.364	<0.0001 ****	12
Spatial Spread (μm)	7.312 ± 0.6111	2.649 ± 0.7526	0.0005 ***	12
Duration (ms)	3.487 ± 0.1955	1.331 ± 0.415	0.0007 ***	12
Area (Distance μm*s)	1.679 ± 0.1734	0.5255 ± 0.166	0.0006 ***	12

**Calcium Signal Parameter ICC-DMP**	**AT7867 (10μM)**	**AT7867 + Secretin**		
	Mean-SEM	Mean-SEM	P value	N
Frequency (Ca^{2+} transients/30s)	14.91 ± 2.897	11.88 ± 2.814	0.0312 *	12
Spatial Spread (μm)	7.523 ± 1.29	8.199 ± 1.282	0.4534 ns	12
Duration (ms)	3.755 ± 0.2818	4.185 ± 0.3422	0.1037 ns	12
Area (Distance μm*s)	2.197 ± 0.4111	2.345 ± 0.3149	0.6052 ns	12

P value < 0.05 are represented by three asterisks (*). P value < 0.001 are represented by three asterisks (***). P value < 0.0001 are represented by four asterisks (****). No significance refers to ns. N refers to the number of animals.

Appendix Table S13: **Summary analysis of murine contraction parameters (frequency, AUC and amplitude) in response to ESI-05 and secretin.**

**Contractile parameter -Murine Small intestine**	**Control**	**Secretin (100nM)**		
	Mean-SEM	Mean-SEM	P value	N
Frequency	33.22 ± 2.184	30.44 ± 2.789	0.1934 ns	5
AUC (mN*min)	100%	54.24% ± 7.536	0.0003 ***	5
Peak amplitude (mN)	100%	76.52% ± 6.034	0.0046 **	5

**Contractile parameter -Murine Small intestine**	**ESI-05 (10µM)**	**ESI-05+Secretin**		
	Mean-SEM	Mean-SEM	P value	N
Frequency	34.29 ± 1.755	33.71 ± 1.835	0.5081 ns	4
AUC (mN*min)	100%	66.5% ± 4.608	0.0003 **	4
Peak amplitude (mN)	100%	82.47% ± 4.189	0.0058 **	4

**Contractile parameter -Murine Small intestine**	**Control**	**ESI-05**		
	Mean-SEM	Mean-SEM	P value	N
Frequency	32.14 ± 1.64	34 ± 1.813	0.3228 ns	4
AUC (mN*min)	100%	200.1% ± 55.64	0.1222 ns	4
Peak amplitude (mN)	100%	142% ± 22.43	0.1105 ns	4

P value < 0.01 are represented by four asterisks (**). P value < 0.001 are represented by three asterisks (***). No significance refers to ns. N refers to the number of animals.

Appendix Table S14: **Summary analysis of Ca^{2+} transient parameters (frequency, spatial spread, duration and event area) of ICC-DMP in the presence of ESI-05 and secretin.**

**Calcium Signal Parameter ICC-DMP**	**Control**	**Secretin (100nM)**		
	Mean-SEM	Mean-SEM	P value	N
Frequency (Ca^{2+} transients/30s)	21.64 ± 3.651	0.8182 ± 0.4635	0.0004 ***	5
Spatial Spread (µm)	9.092 ± 1.327	1.914 ± 0.8786	0.0008 ***	5
Duration (ms)	3.238 ± 0.1852	0.8199 ± 0.3509	0.0003 ***	5
Area (Distance µm*s)	1.955 ± 0.2597	0.2867 ± 0.1339	0.0003 ***	5
**Calcium Signal Parameter ICC-DMP**	**ESI-05 (10µM)**	**ESI-05+Secretin**		
	Mean-SEM	Mean-SEM	P value	N
Frequency (Ca^{2+} transients/30s)	16 ± 2.431	3.273 ± 1.695	0.0003 ***	5
Spatial Spread (µm)	13.23 ± 1.527	3.679 ± 1.49	0.0013 **	5
Duration (ms)	3.241 ± 0.1818	1.031 ± 0.3768	<0.0001 ****	5
Area (Distance µm*s)	2.69 ± 0.3616	0.559 ± 0.2438	0.0001 ***	5

P value < 0.01 are represented by four asterisks (**). P value < 0.001 are represented by three asterisks (***). P value < 0.0001 are represented by four asterisks (****). No significance refers to ns. N refers to the number of animals.
