# Supplementary material for: Secretin targets interstitial cells of Cajal to regulate intestinal contractions
Source: EMBO Rep. 2025 Nov 6;26(23):6015–43. doi: 10.1038/s44319-025-00623-1 (PMC12678811; doi:10.1038/s44319-025-00623-1)
Supplement: Supplementary file 3 — Movie EV1 [file 44319_2025_623_MOESM3_ESM.zip › Movie EV1/Movie EV1 legend.docx]

**Movie EV1: Secretin inhibit ICC-DMP Ca^2+^ transients in the small intestine.**

The movie captures subcellular Ca^2+^ transients in ICC-DMP in the small intestine at high resolution imaged with a 60× objective. Ca^2+^ signals were monitored using the genetically encoded Ca^2+^ indicator GCaMP6f exclusively expressed in ICC. The movie shows typical spindle-shaped ICC-DMP. The scale bar (white) is 10 µm. In the lower panel, a spatio-temporal map (STMap) of ICC-DMP Ca^2+^ transients are shown, where the x-axis represents time, and the y-axis represents space. Application of secretin (100 nM) inhibited Ca^2+^ transients in ICC-DMP.
