# Supplementary figures and images for: Secretin targets interstitial cells of Cajal to regulate intestinal contractions
Source: EMBO Rep. 2025 Nov 6;26(23):6015–43. doi: 10.1038/s44319-025-00623-1 (PMC12678811; doi:10.1038/s44319-025-00623-1)

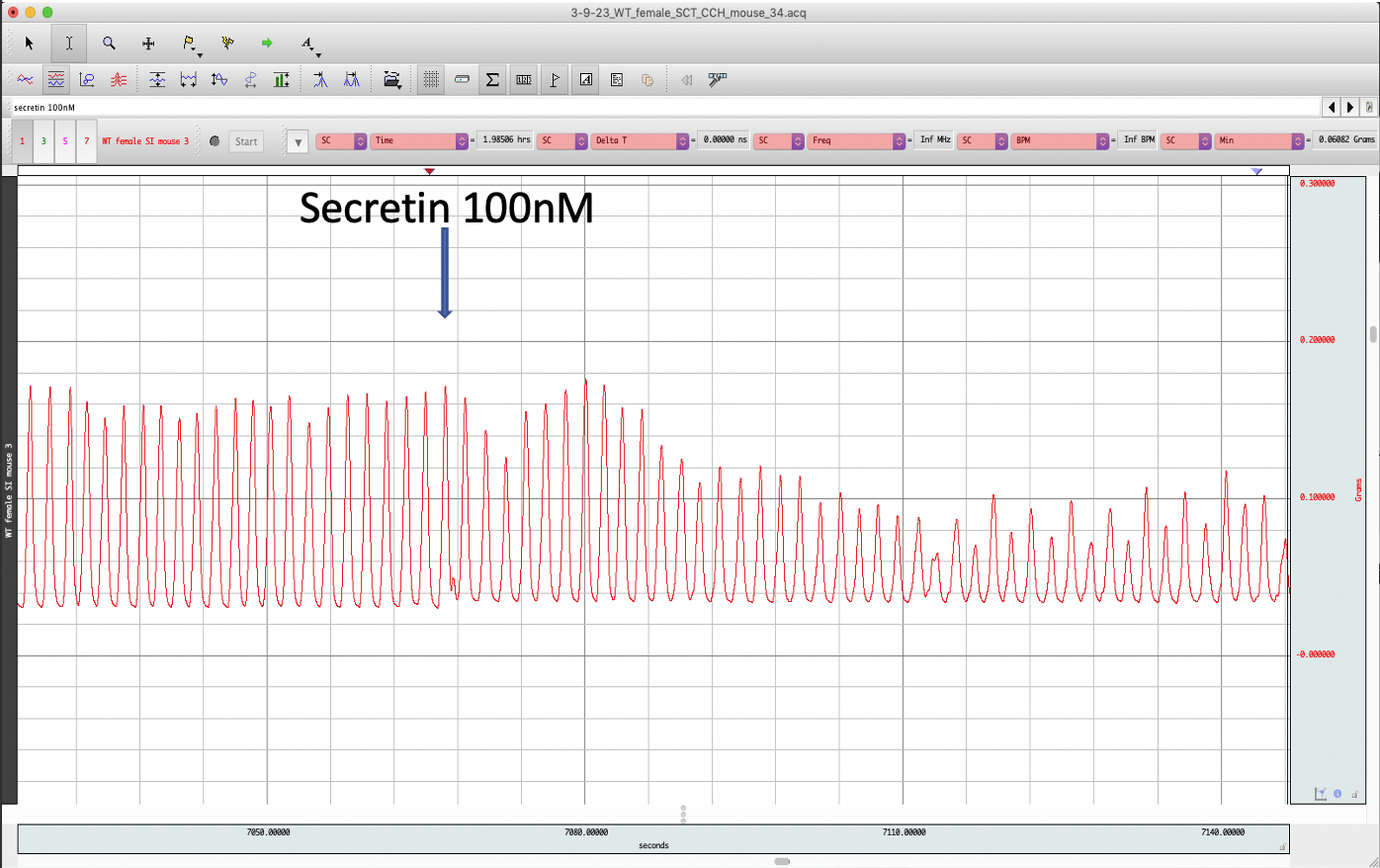

Supplement: Supplementary file 4 — Source data Fig. 1 [file 44319_2025_623_MOESM4_ESM.zip › Figure 1/1 A-H/1A/Trace1_3-9-23_WT_female_SCT_mouse_34_.png]

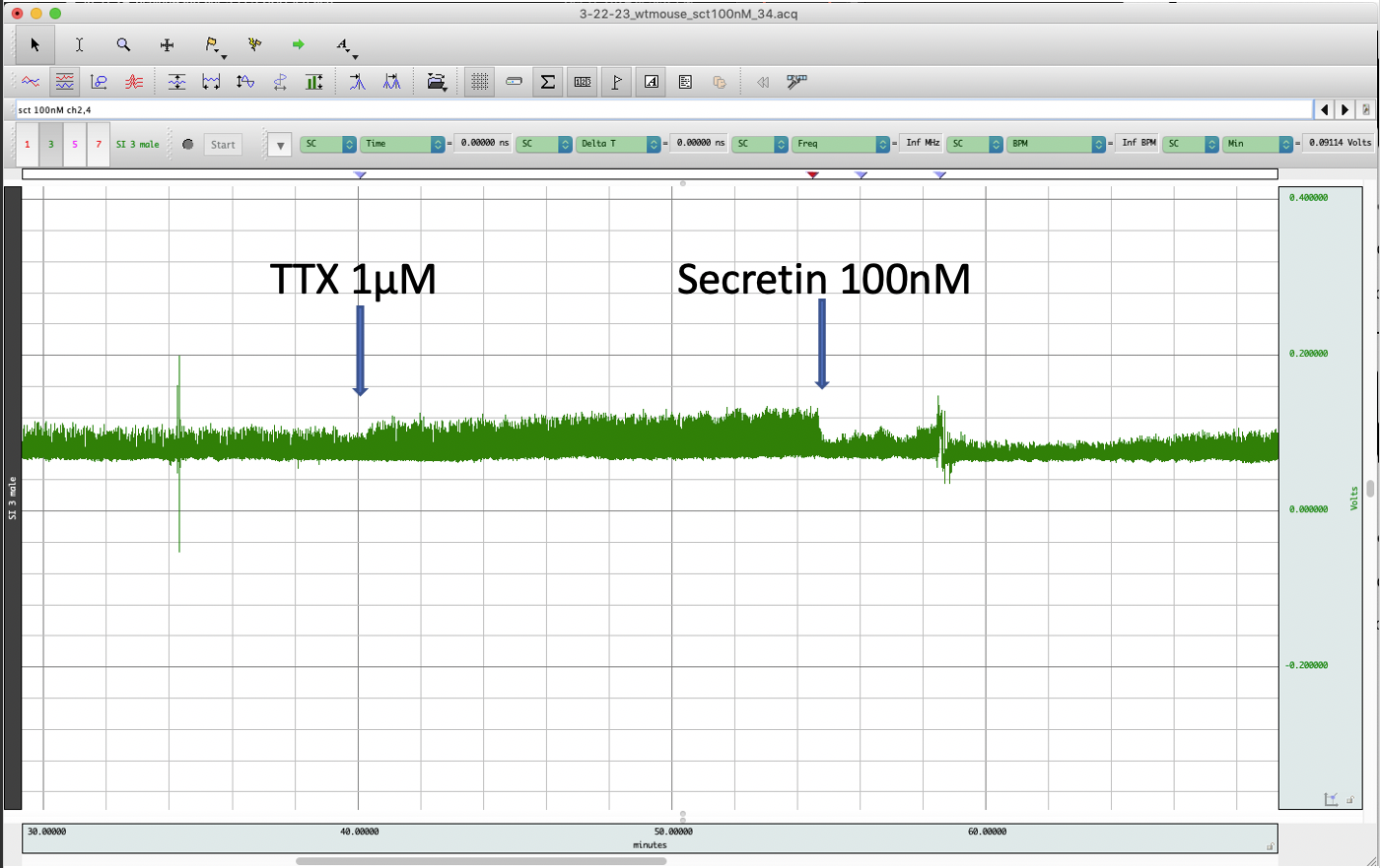

Supplement: Supplementary file 4 — Source data Fig. 1 [file 44319_2025_623_MOESM4_ESM.zip › Figure 1/1 A-H/1E/Trace2_3-22-23_wtmouse_sct100nM_34.png]

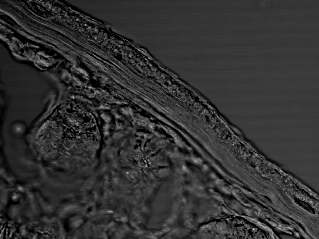

Supplement: Supplementary file 5 — Source data Fig. 2 [file 44319_2025_623_MOESM5_ESM.zip › Figure 2/2A-H/2A/TD_WT_Jejunum slide7-SCTR_RNA594- mSCFR488_20x001.nd2 - C=3-1CROP-final copy.tif]

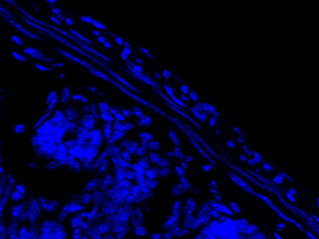

Supplement: Supplementary file 5 — Source data Fig. 2 [file 44319_2025_623_MOESM5_ESM.zip › Figure 2/2A-H/2B/DAPI_WT_Jejunum slide7-SCTR_RNA594- mSCFR488_20x001.nd2 - C=0-CROP-final copy.tif]

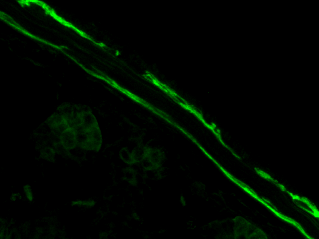

Supplement: Supplementary file 5 — Source data Fig. 2 [file 44319_2025_623_MOESM5_ESM.zip › Figure 2/2A-H/2C/GREEN_mscfr_WT_Jejunum slide7-SCTR_RNA594- mSCFR488_20x001.nd2 - C=1-CROP-final copy.tif]

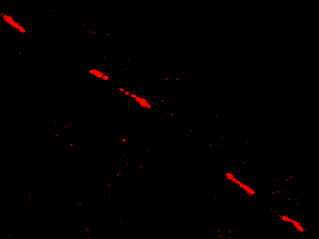

Supplement: Supplementary file 5 — Source data Fig. 2 [file 44319_2025_623_MOESM5_ESM.zip › Figure 2/2A-H/2D/RED_sctr_WT_Jejunum slide7-SCTR_RNA594- mSCFR488_20x001.nd2 - C=2-1CROP-final copy.tif]

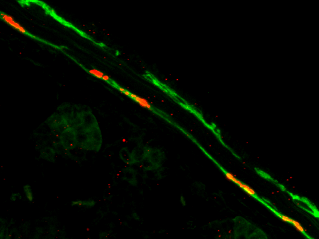

Supplement: Supplementary file 5 — Source data Fig. 2 [file 44319_2025_623_MOESM5_ESM.zip › Figure 2/2A-H/2E/WT_Jejunum slide7-20x_RG-CROP-final copy.tif]

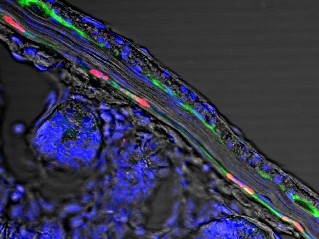

Supplement: Supplementary file 5 — Source data Fig. 2 [file 44319_2025_623_MOESM5_ESM.zip › Figure 2/2A-H/2F/WT_Jejunum slide7-20x_allmerge_edited-1CRPOP-final copy.tif]

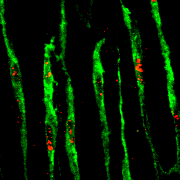

Supplement: Supplementary file 5 — Source data Fig. 2 [file 44319_2025_623_MOESM5_ESM.zip › Figure 2/2A-H/2G/DMP_merge_red_greenonly_20x_5-2-23_slide4 copy.tif]

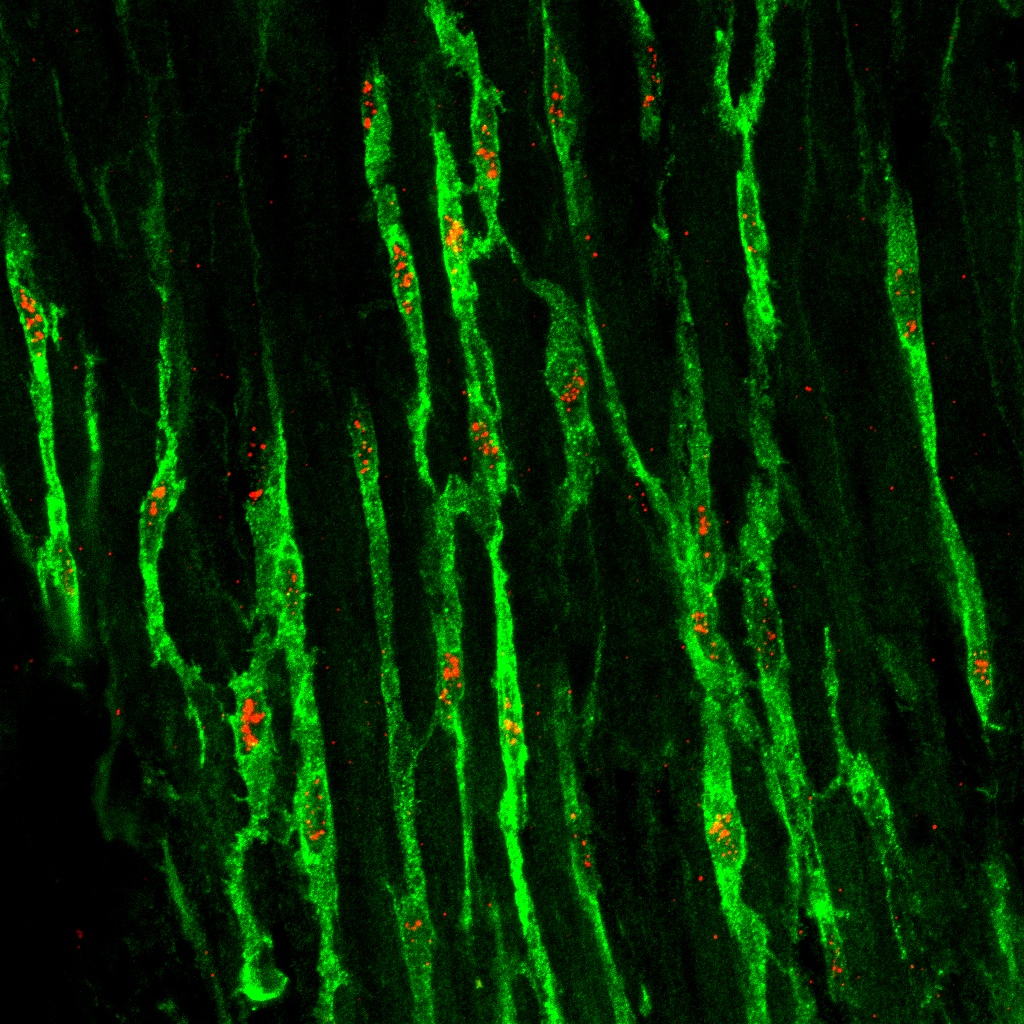

Supplement: Supplementary file 5 — Source data Fig. 2 [file 44319_2025_623_MOESM5_ESM.zip › Figure 2/2A-H/2G/Merge_redGreen_MAX_5-3-23RNAscopeSI_CKIT488_SCTRRNA594_60X_dmpONLY_flat.tif]

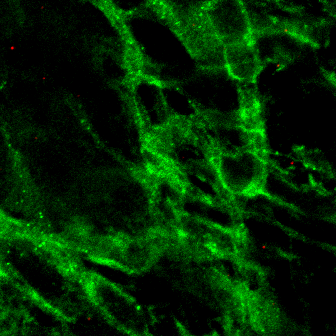

Supplement: Supplementary file 5 — Source data Fig. 2 [file 44319_2025_623_MOESM5_ESM.zip › Figure 2/2A-H/2H/Merge_redGreen_MAX_5-3-23RNAscopeSI_CKIT488_SCTRRNA594_60X.nd2 - C=0-1_MY.tif]

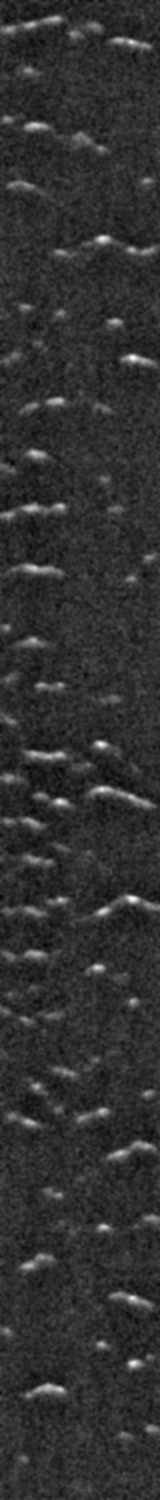

Supplement: Supplementary file 6 — Source data Fig. 3 [file 44319_2025_623_MOESM6_ESM.zip › Figure 3/3B-C/STMAPS_secretin_TTX_DMP/GC6kit_Small Intestine_100nM Nicardipine_1uM TTX 15 min_20x_STMapIM1 copy.tif]

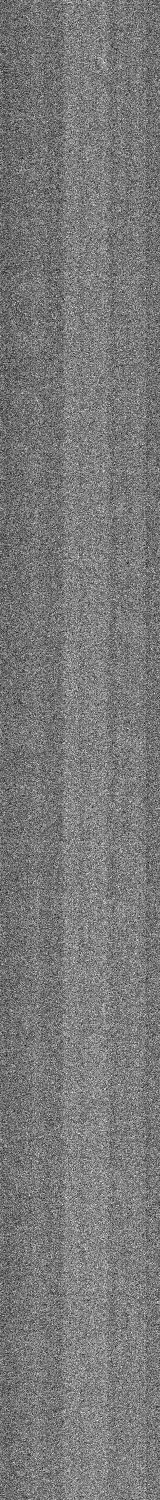

Supplement: Supplementary file 6 — Source data Fig. 3 [file 44319_2025_623_MOESM6_ESM.zip › Figure 3/3B-C/STMAPS_secretin_TTX_DMP/GC6kit_Small Intestine_100nM Nicardipine_1uM TTX _Secritin 100nM 3 Min_20x_STMapIM1 copy.tif]

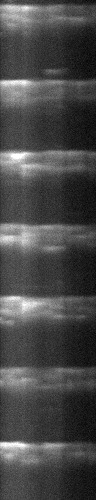

Supplement: Supplementary file 6 — Source data Fig. 3 [file 44319_2025_623_MOESM6_ESM.zip › Figure 3/3I-J/STMAPS_secretin_TTX_MY/12-11-24_AREA3_40x_kitGC_duod_IMMY_TTX1uM_Cntrl_STMapMY1-500frames.tif]

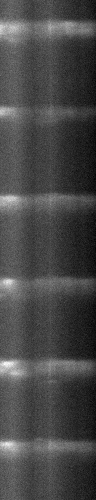

Supplement: Supplementary file 6 — Source data Fig. 3 [file 44319_2025_623_MOESM6_ESM.zip › Figure 3/3I-J/STMAPS_secretin_TTX_MY/12-11-24_AREA3_40x_kitGC_duod_IMMY_TTX1uM_secretin100nM5min_STMapMY1-500frames.tif]

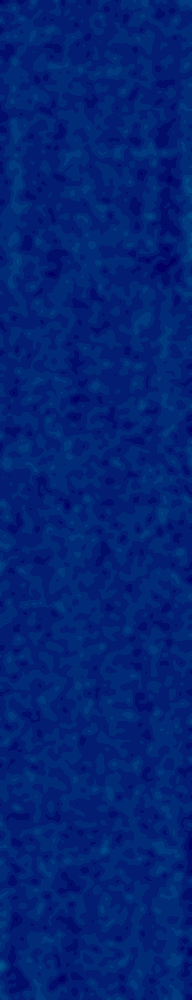

Supplement: Supplementary file 7 — Source data Fig. 4 [file 44319_2025_623_MOESM7_ESM.zip › Figure 4/4A-B/STMAPS_3Hz_EFS/COLOR_11-6-23_40x_gc6_IM_nicard200nM_EFS3hzcontinuous_20secCNTRL_sct100nM_5min_STMapCell1-1000frames copy.tif]

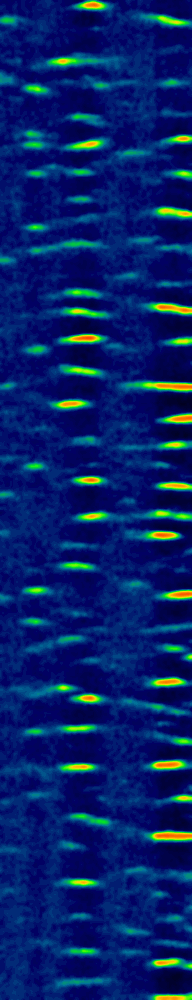

Supplement: Supplementary file 7 — Source data Fig. 4 [file 44319_2025_623_MOESM7_ESM.zip › Figure 4/4A-B/STMAPS_3Hz_EFS/COLOR_11-6-23_40x_gc6_IM_nicard200nM_EFS3hzcontinuous_20secCNTRL_STMapcell1-1000frames copy.tif]

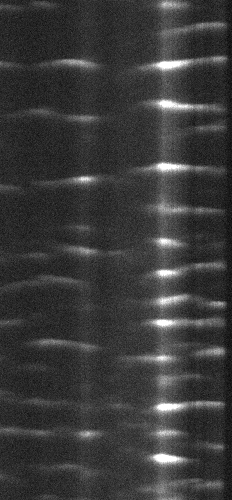

Supplement: Supplementary file 7 — Source data Fig. 4 [file 44319_2025_623_MOESM7_ESM.zip › Figure 4/4G-H/STMAPS_10Hz_EFS/8-14-24_40x_gc6kit_dmp_mrs25001uM_lnna100uM_2xstim10sec_1min10sec_10hz10sec_secondBest_STMap1-1after copy.tif]

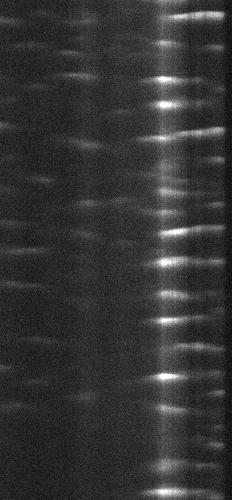

Supplement: Supplementary file 7 — Source data Fig. 4 [file 44319_2025_623_MOESM7_ESM.zip › Figure 4/4G-H/STMAPS_10Hz_EFS/8-14-24_40x_gc6kit_dmp_mrs25001uM_lnna100uM_2xstim10sec_1min10sec_10hz10sec_secondBest_STMap1-1before copy.tif]

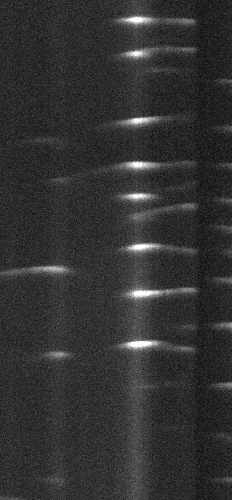

Supplement: Supplementary file 7 — Source data Fig. 4 [file 44319_2025_623_MOESM7_ESM.zip › Figure 4/4G-H/STMAPS_10Hz_EFS/8-14-24_40x_gc6kit_dmp_mrs25001uM_lnna100uM_2xstim10sec_1min10sec_10hz10sec_secretin100nM3min_STMap1-1after copy.tif]

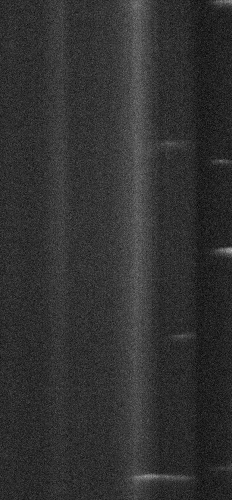

Supplement: Supplementary file 7 — Source data Fig. 4 [file 44319_2025_623_MOESM7_ESM.zip › Figure 4/4G-H/STMAPS_10Hz_EFS/8-14-24_40x_gc6kit_dmp_mrs25001uM_lnna100uM_2xstim10sec_1min10sec_10hz10sec_secretin100nM3min_STMap1-1before copy.tif]

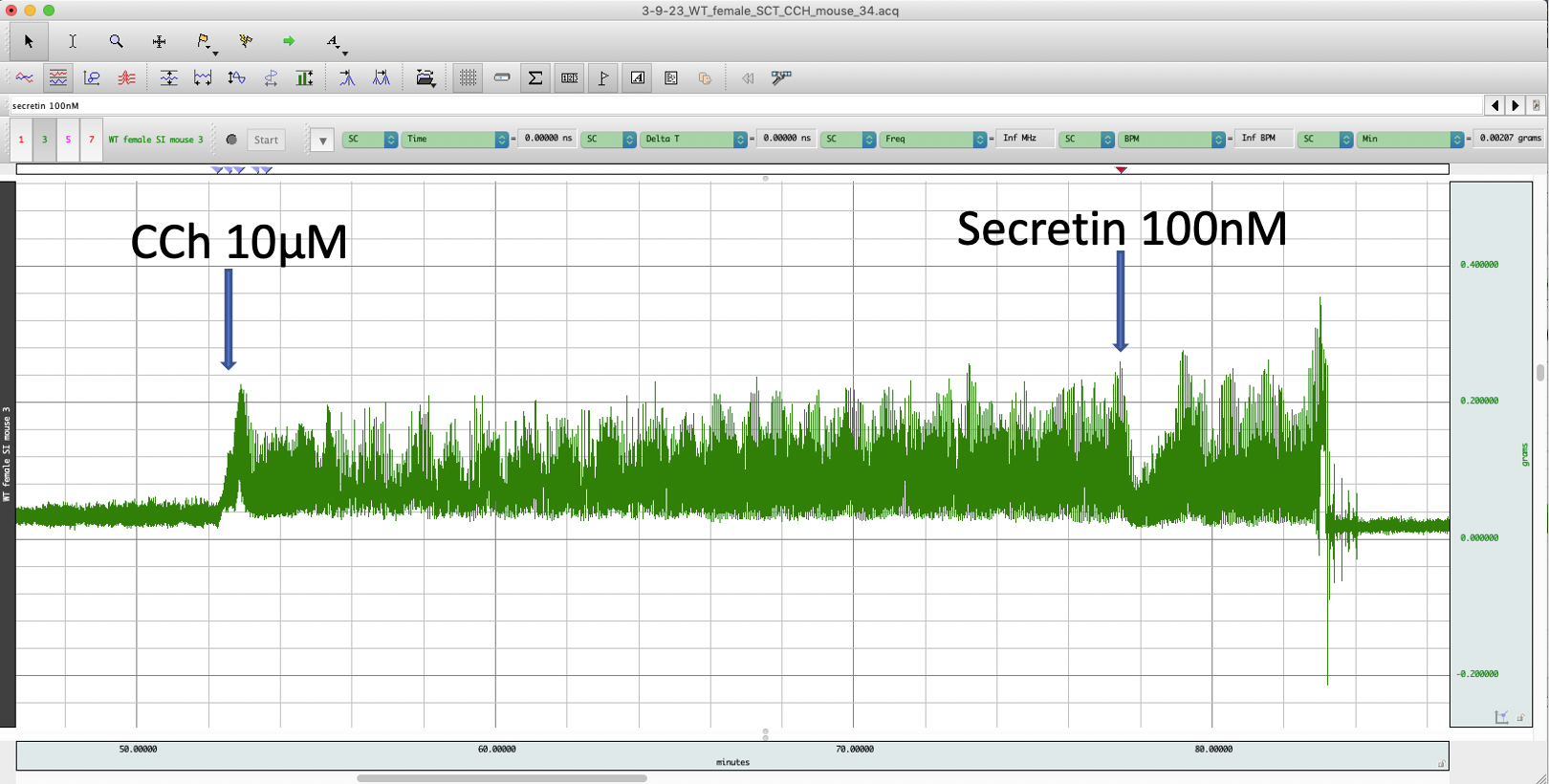

Supplement: Supplementary file 8 — Source data Fig. 5 [file 44319_2025_623_MOESM8_ESM.zip › Figure 5/5A-H/5A/trace1_3-9-23_WT_female_SCT_CCH_mouse_34.png]

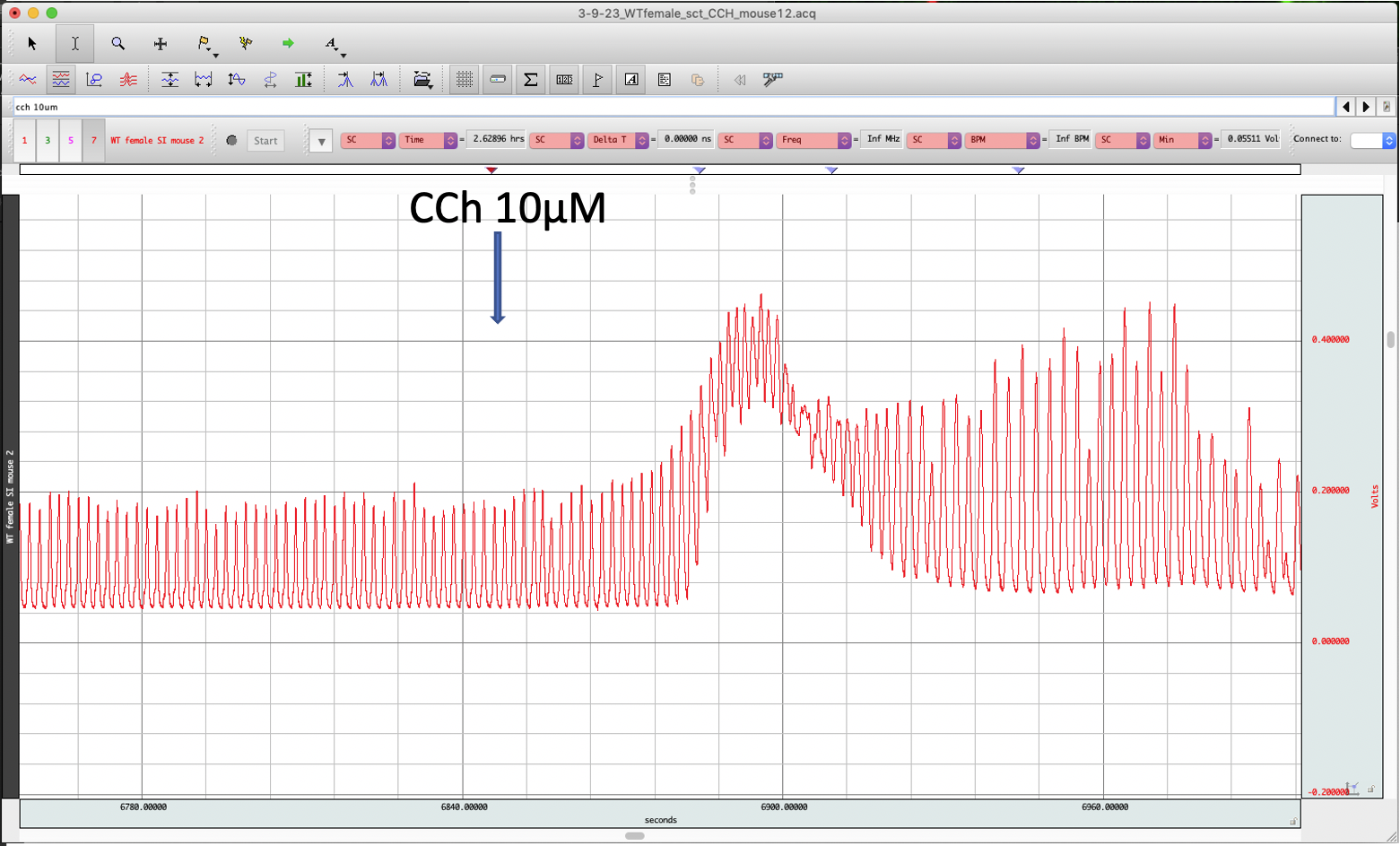

Supplement: Supplementary file 8 — Source data Fig. 5 [file 44319_2025_623_MOESM8_ESM.zip › Figure 5/5A-H/5E/justCCh_3-9-23.png]

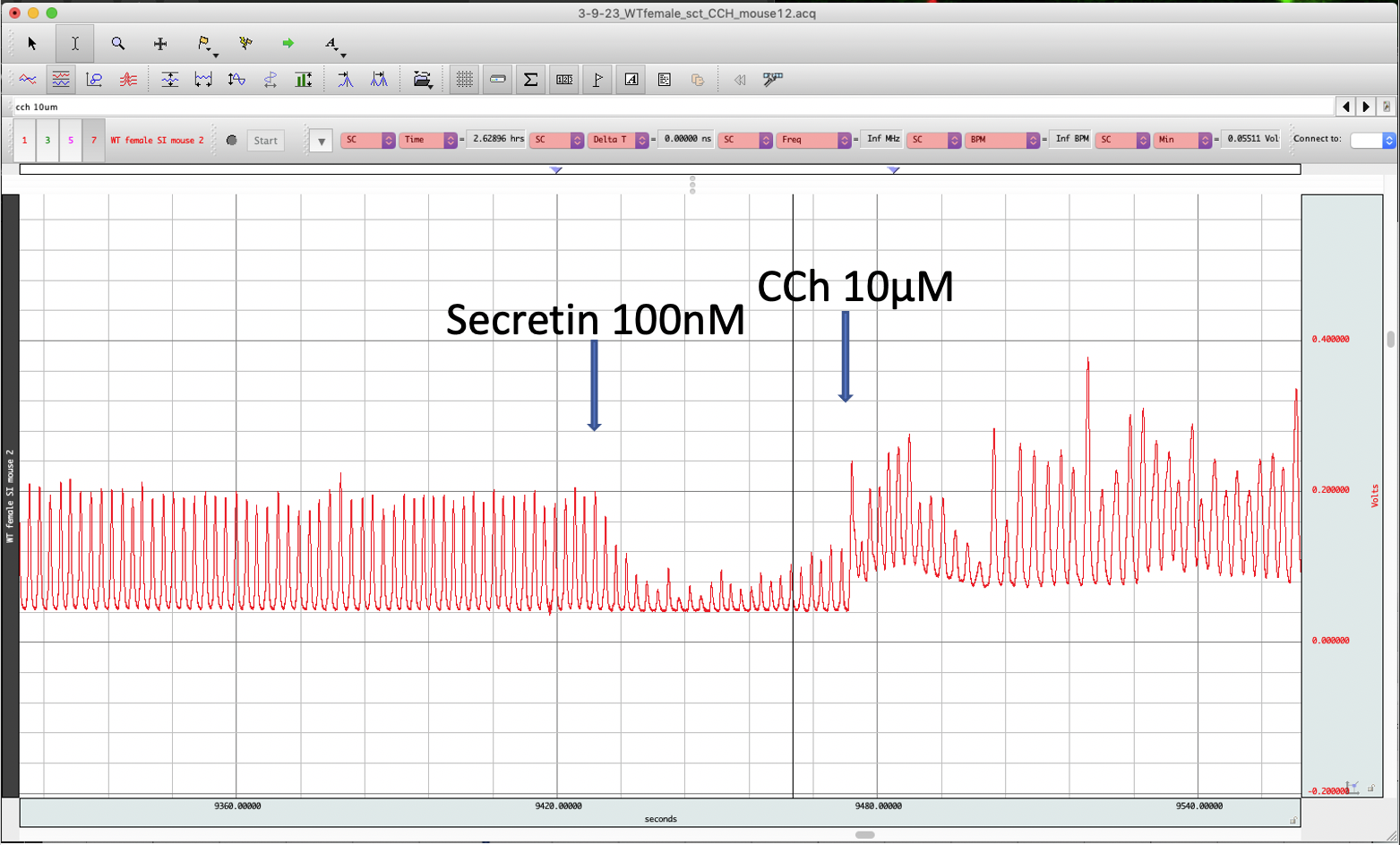

Supplement: Supplementary file 8 — Source data Fig. 5 [file 44319_2025_623_MOESM8_ESM.zip › Figure 5/5A-H/5E/secretin_CCh_3-9-23_.png]

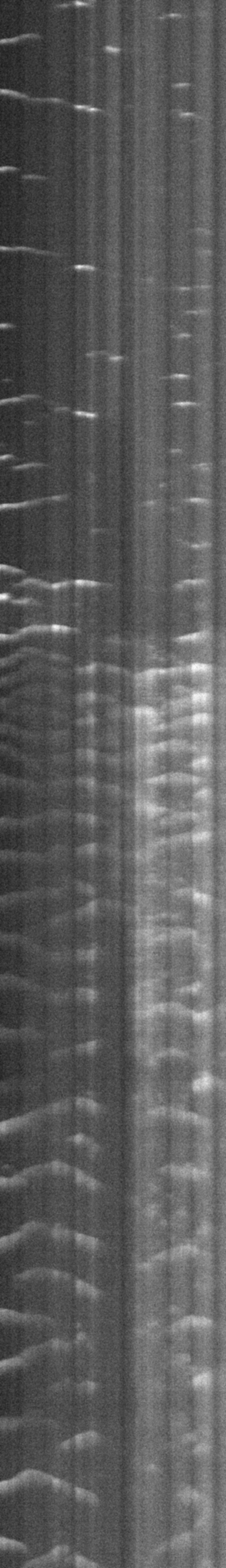

Supplement: Supplementary file 9 — Source data Fig. 6 [file 44319_2025_623_MOESM9_ESM.zip › Figure 6/6B-C/4-9-24_Area4_60x_gc6kit_cagedIp3_SI_dmp_20sCNTRL_10pwrPulse_x10_50sec_repeat_aftersctWASHOUTCNTRL_STMap.tif]

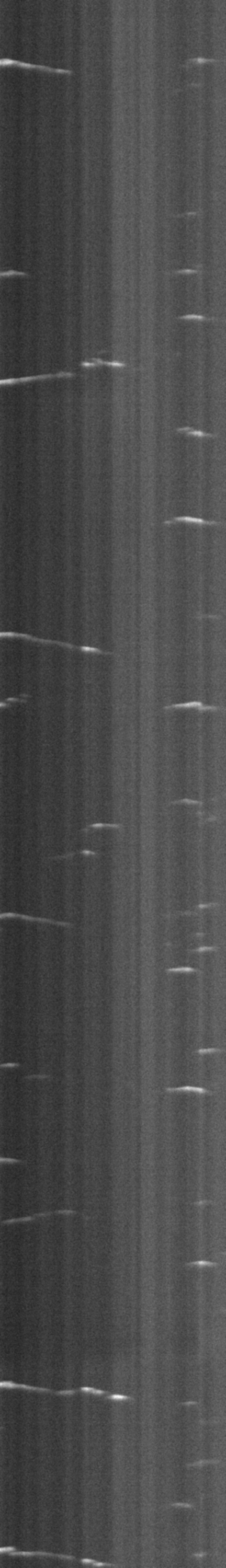

Supplement: Supplementary file 9 — Source data Fig. 6 [file 44319_2025_623_MOESM9_ESM.zip › Figure 6/6B-C/4-9-24_Area4_60x_gc6kit_cagedIp3_SI_dmp_20sCNTRL_10pwrPulse_x10_50sec_repeat_secretin100nm_6min_STMap.tif]

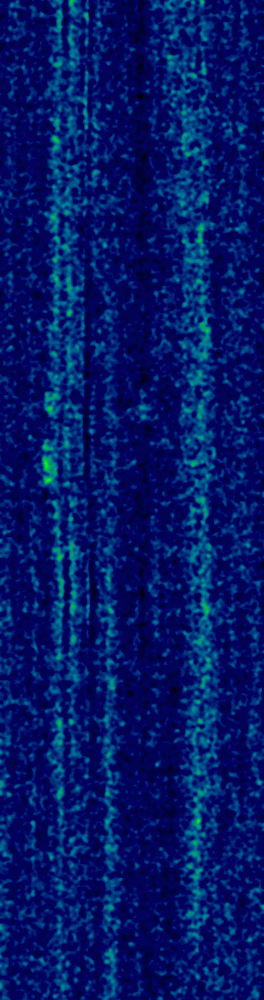

Supplement: Supplementary file 10 — Source data Fig. 7 [file 44319_2025_623_MOESM10_ESM.zip › Figure 7/7A-C/Color8-3-23_KitGc6_40x_SI-nicard200nM_30sCNTRL_sct100nMpuff_IM_STMapcell1-1after.tif]

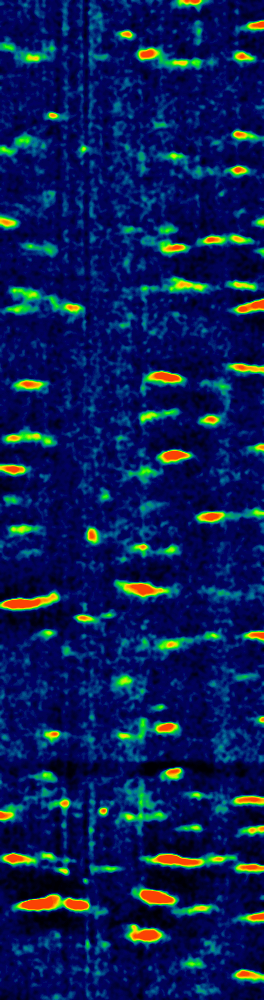

Supplement: Supplementary file 10 — Source data Fig. 7 [file 44319_2025_623_MOESM10_ESM.zip › Figure 7/7A-C/Color8-3-23_KitGc6_40x_SI-nicard200nM_30sCNTRL_sct100nMpuff_IM_STMapcell1-1Cntrl.tif]

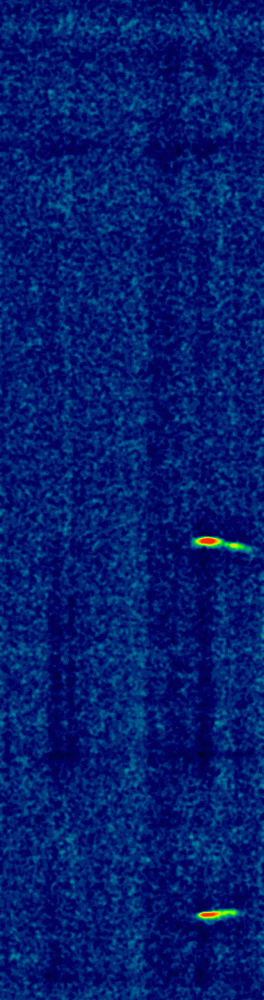

Supplement: Supplementary file 10 — Source data Fig. 7 [file 44319_2025_623_MOESM10_ESM.zip › Figure 7/7A-C/Color8-3-23_KitGc6_40x_SI-ZEROCa_nicard200nM_30sCNTRL_sct100nMpuff_IM_STMapCell1-1After.tif]

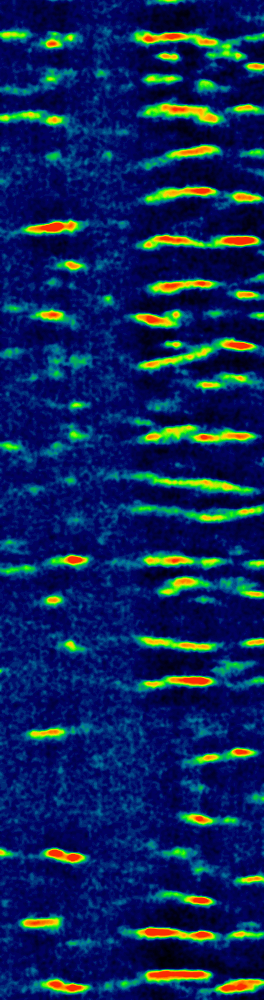

Supplement: Supplementary file 10 — Source data Fig. 7 [file 44319_2025_623_MOESM10_ESM.zip › Figure 7/7A-C/Color8-3-23_KitGc6_40x_SI-ZEROCa_nicard200nM_30sCNTRL_sct100nMpuff_IM_STMapCell1-1before.tif]

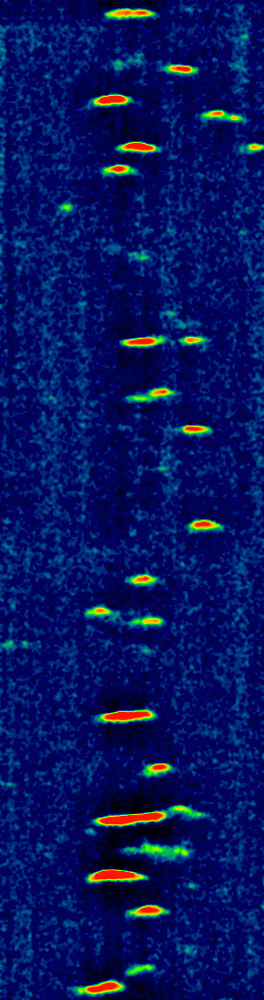

Supplement: Supplementary file 10 — Source data Fig. 7 [file 44319_2025_623_MOESM10_ESM.zip › Figure 7/7I-J/8-3-23_KitGc6_40x_SI-nicard200nM_30sCNTRL_KrebsonlyPuff_STMapcell1-2After.tif]

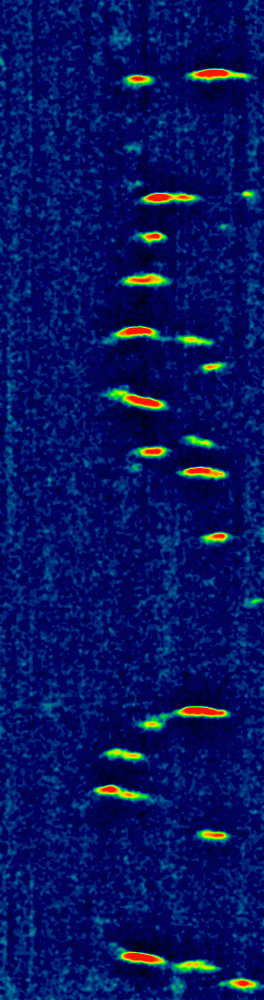

Supplement: Supplementary file 10 — Source data Fig. 7 [file 44319_2025_623_MOESM10_ESM.zip › Figure 7/7I-J/8-3-23_KitGc6_40x_SI-nicard200nM_30sCNTRL_KrebsonlyPuff_STMapcell1-2Before.tif]

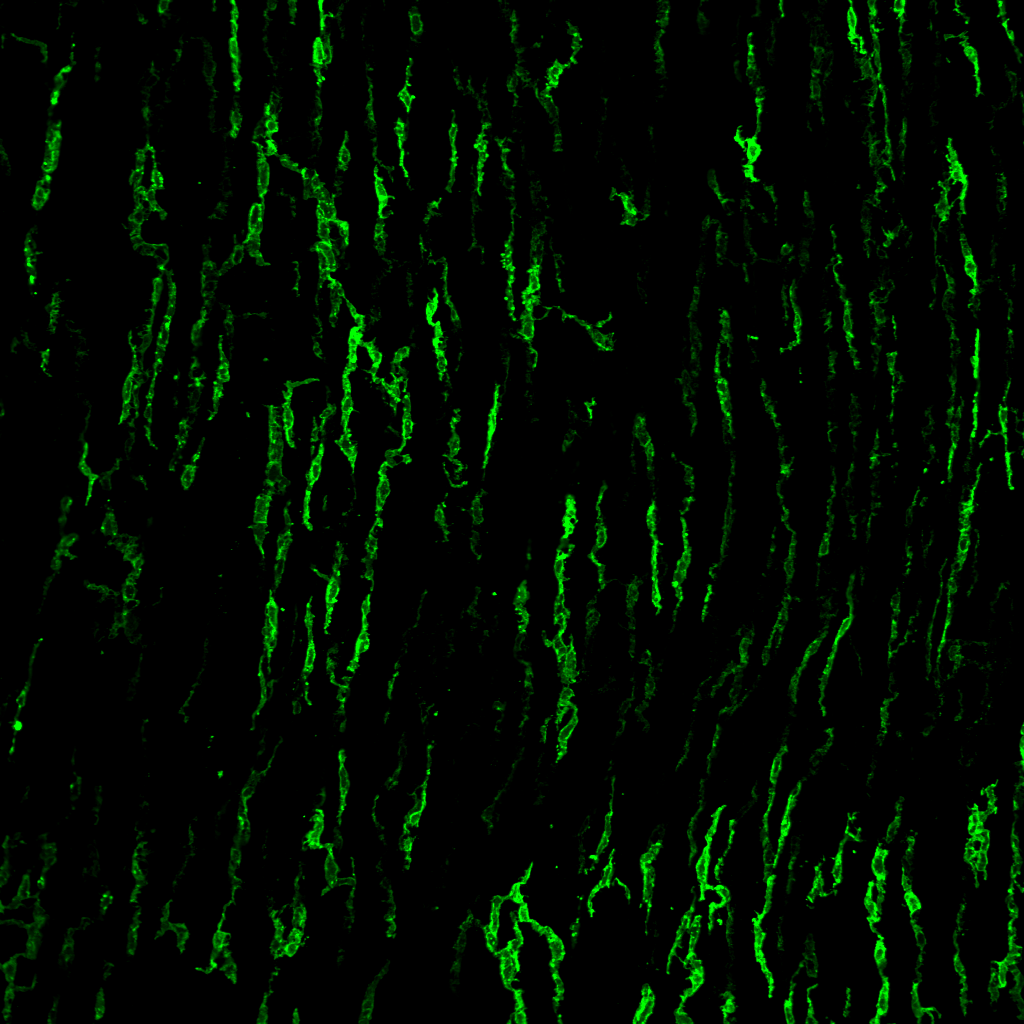

Supplement: Supplementary file 11 — Source data Fig. 8 [file 44319_2025_623_MOESM11_ESM.zip › Figure 8/8A-E/greenMAX_4-19-23_DREADDGskiticre_SIim_GFP488MscFR594_20x.nd2 - C=0 copy.tif]

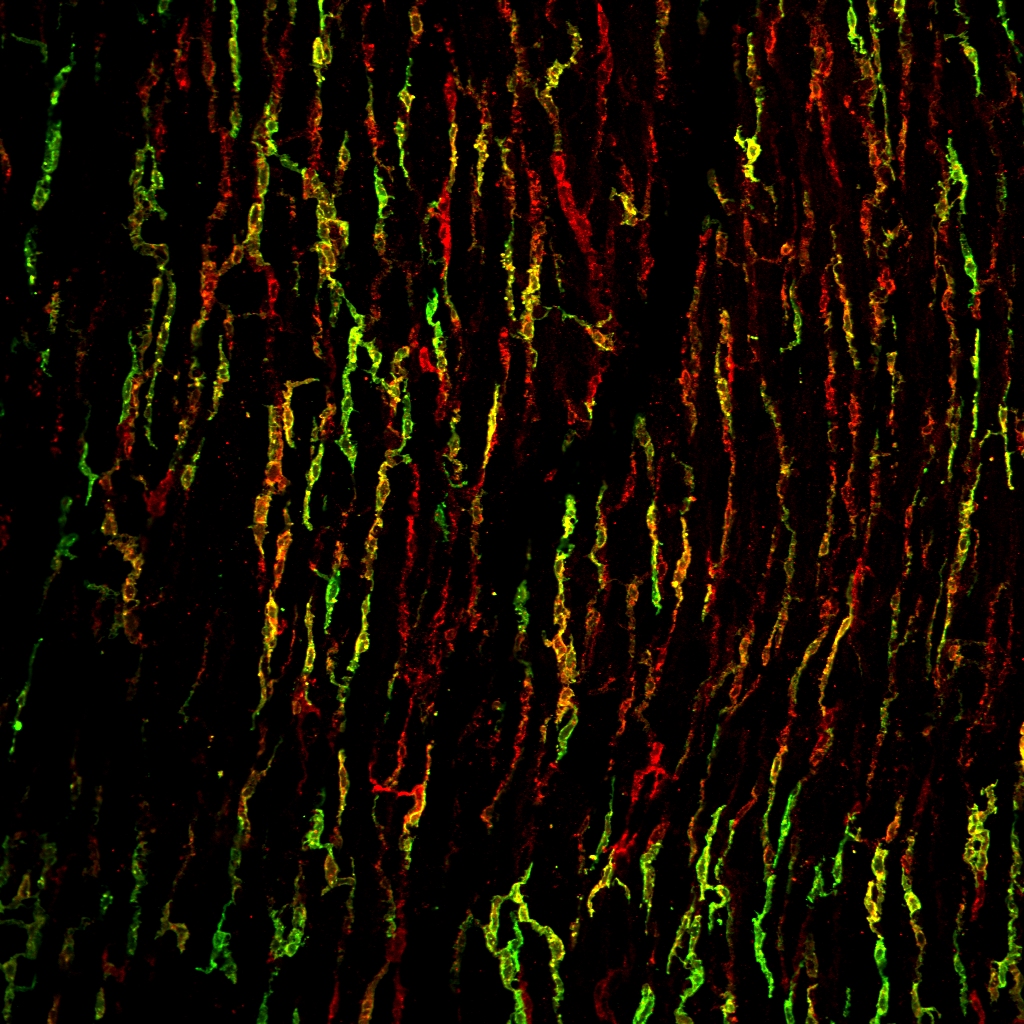

Supplement: Supplementary file 11 — Source data Fig. 8 [file 44319_2025_623_MOESM11_ESM.zip › Figure 8/8A-E/MERGE_4-19-23_DREADDGskiticre_SIim_GFP488MscFR594_20x copy.tif]

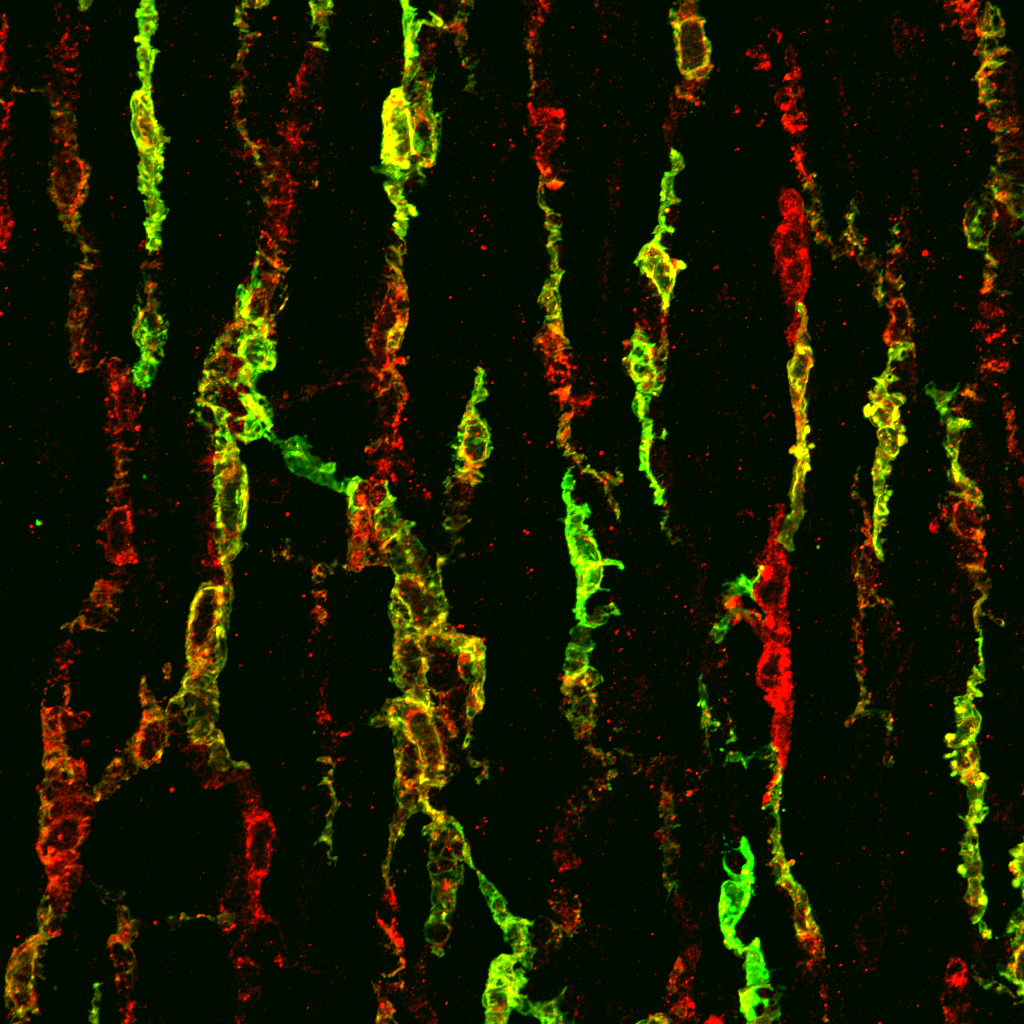

Supplement: Supplementary file 11 — Source data Fig. 8 [file 44319_2025_623_MOESM11_ESM.zip › Figure 8/8A-E/MERGE_4-19-23_DREADDGskiticre_SIim_GFP488MscFR594_60x copy.tif]

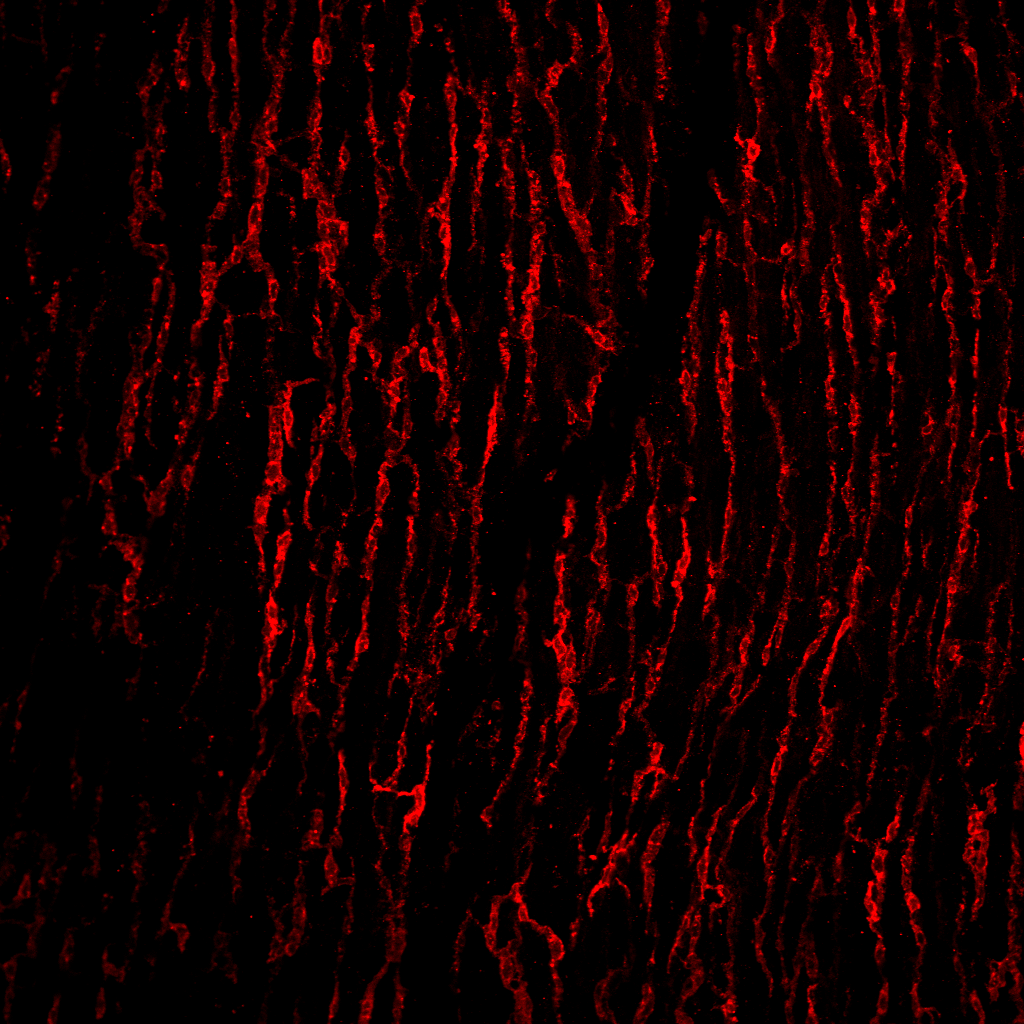

Supplement: Supplementary file 11 — Source data Fig. 8 [file 44319_2025_623_MOESM11_ESM.zip › Figure 8/8A-E/redMAX_4-19-23_DREADDGskiticre_SIim_GFP488MscFR594_20x.nd2 - C=1 copy.tif]

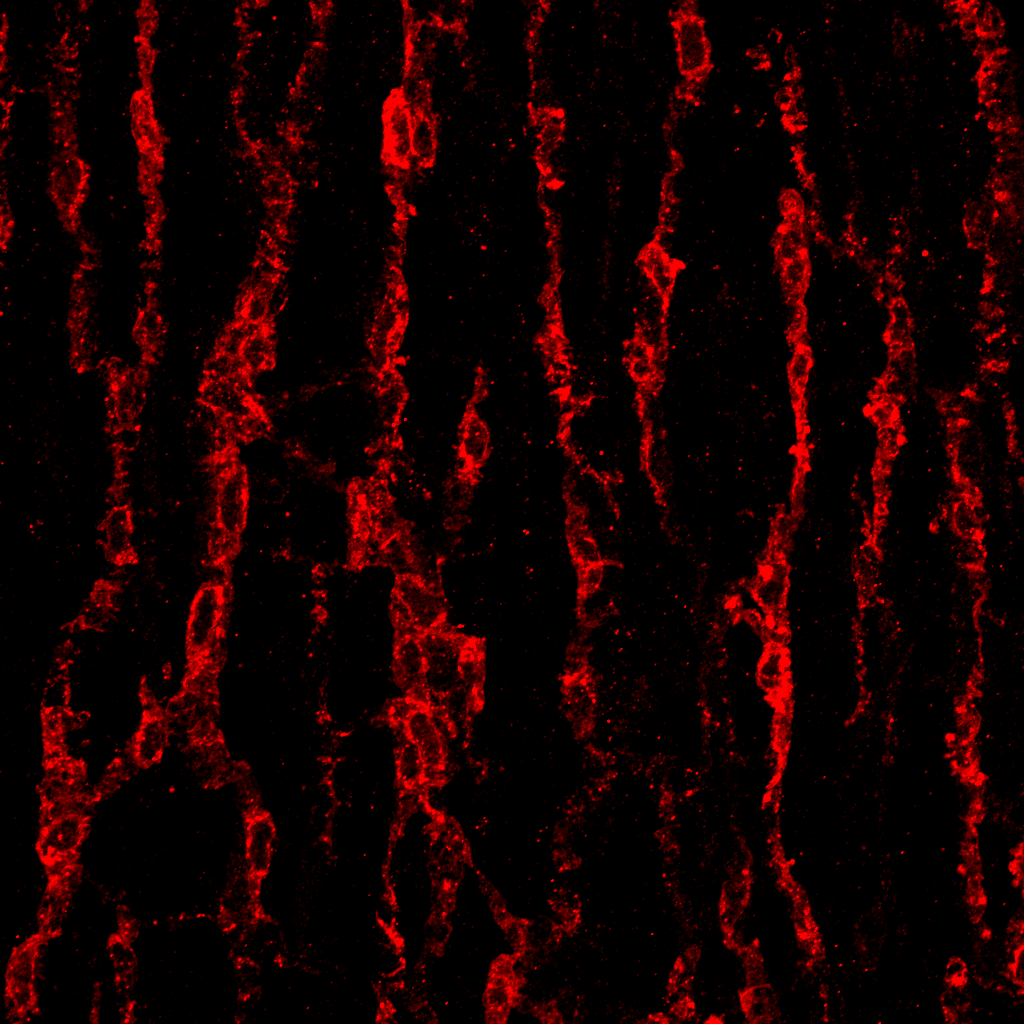

Supplement: Supplementary file 11 — Source data Fig. 8 [file 44319_2025_623_MOESM11_ESM.zip › Figure 8/8A-E/redMAX_4-19-23_DREADDGskiticre_SIim_GFP488MscFR594_60x.nd2 - C=1 copy.tif]

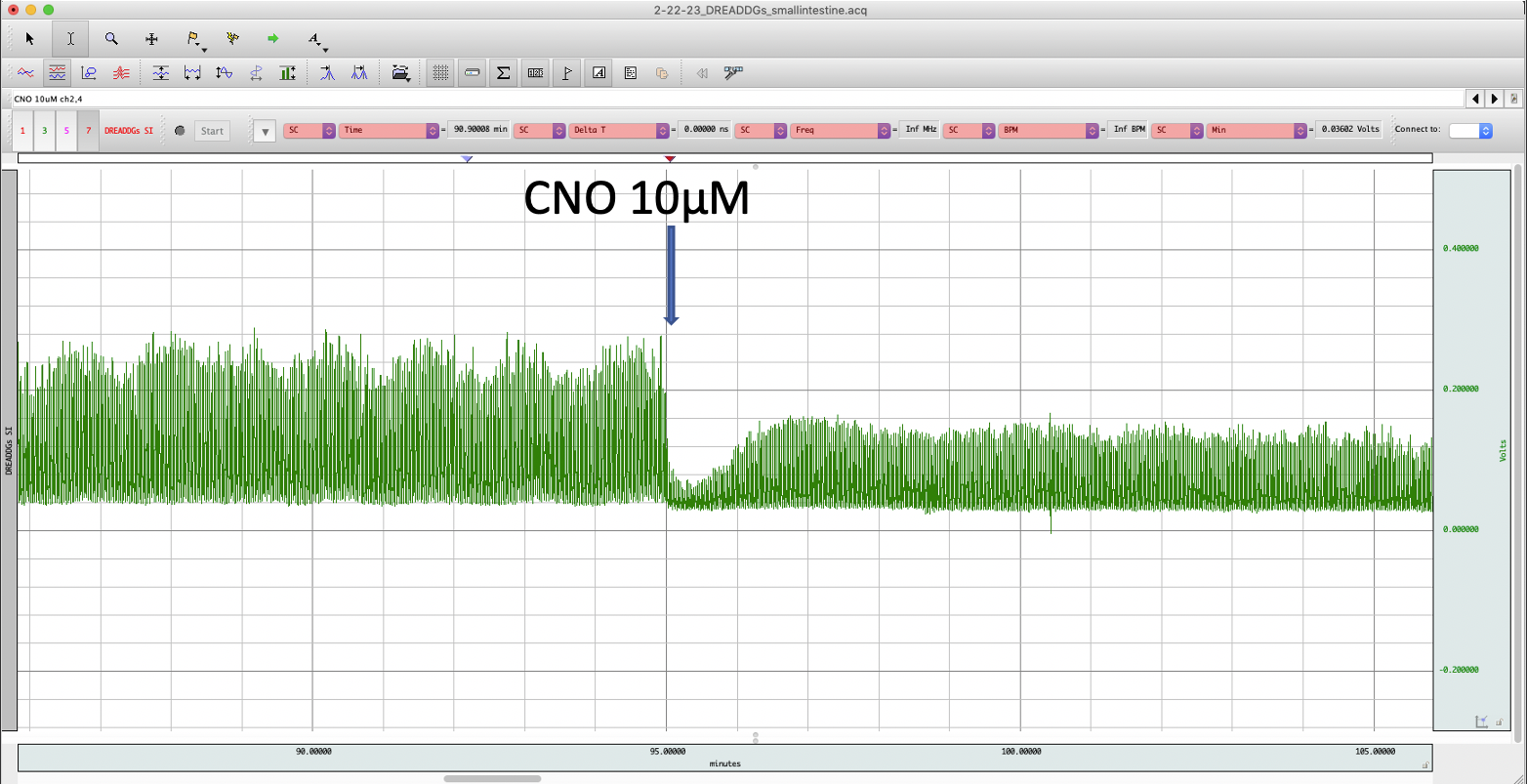

Supplement: Supplementary file 11 — Source data Fig. 8 [file 44319_2025_623_MOESM11_ESM.zip › Figure 8/8F-H/trace_2-22-23_DREADDGs_smallintestine.png]

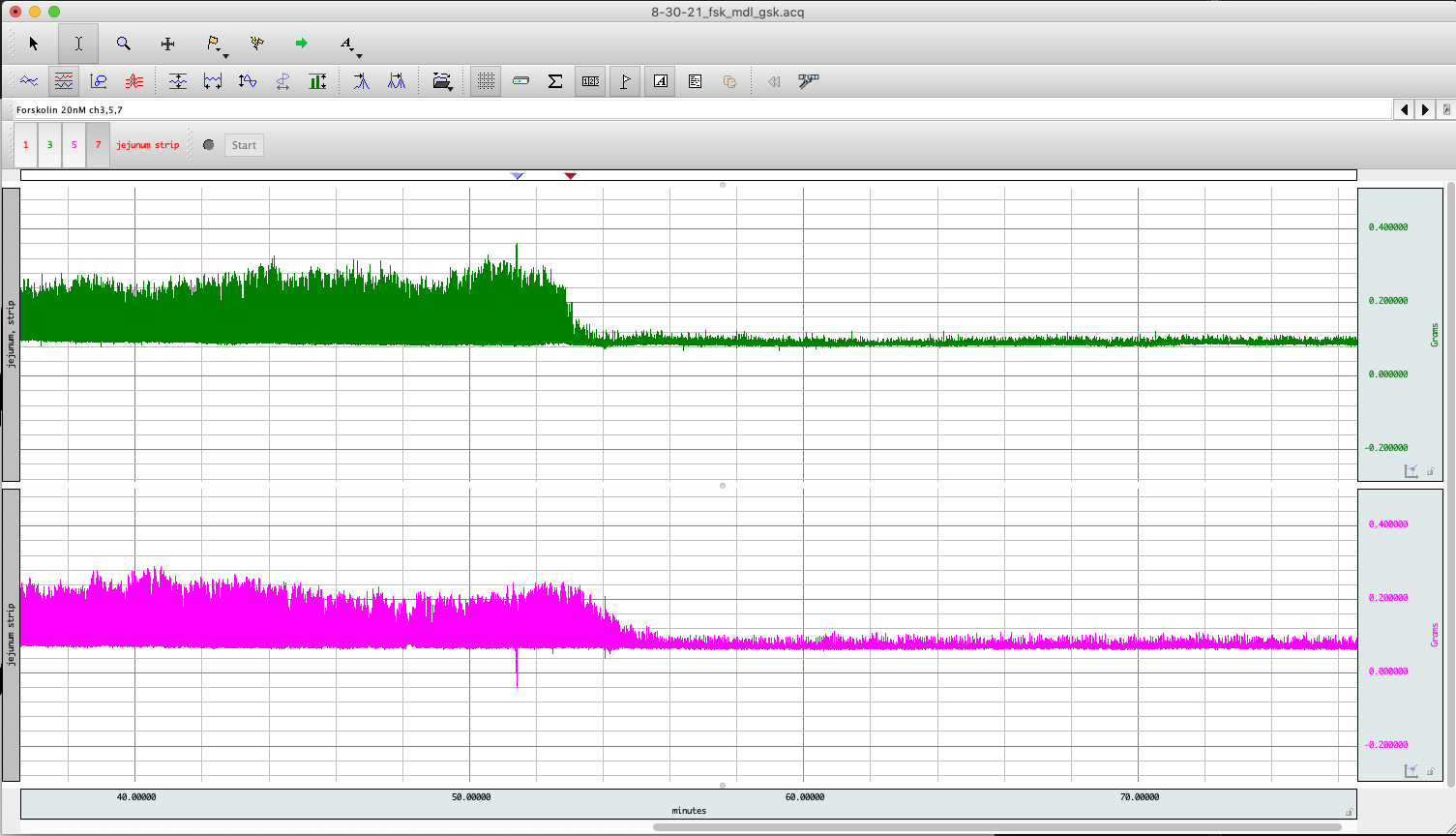

Supplement: Supplementary file 12 — Source data Fig. 9 [file 44319_2025_623_MOESM12_ESM.zip › Figure 9/9D-L/9A-C/traces_8-30-21_fsk_mdl_gsk.png]

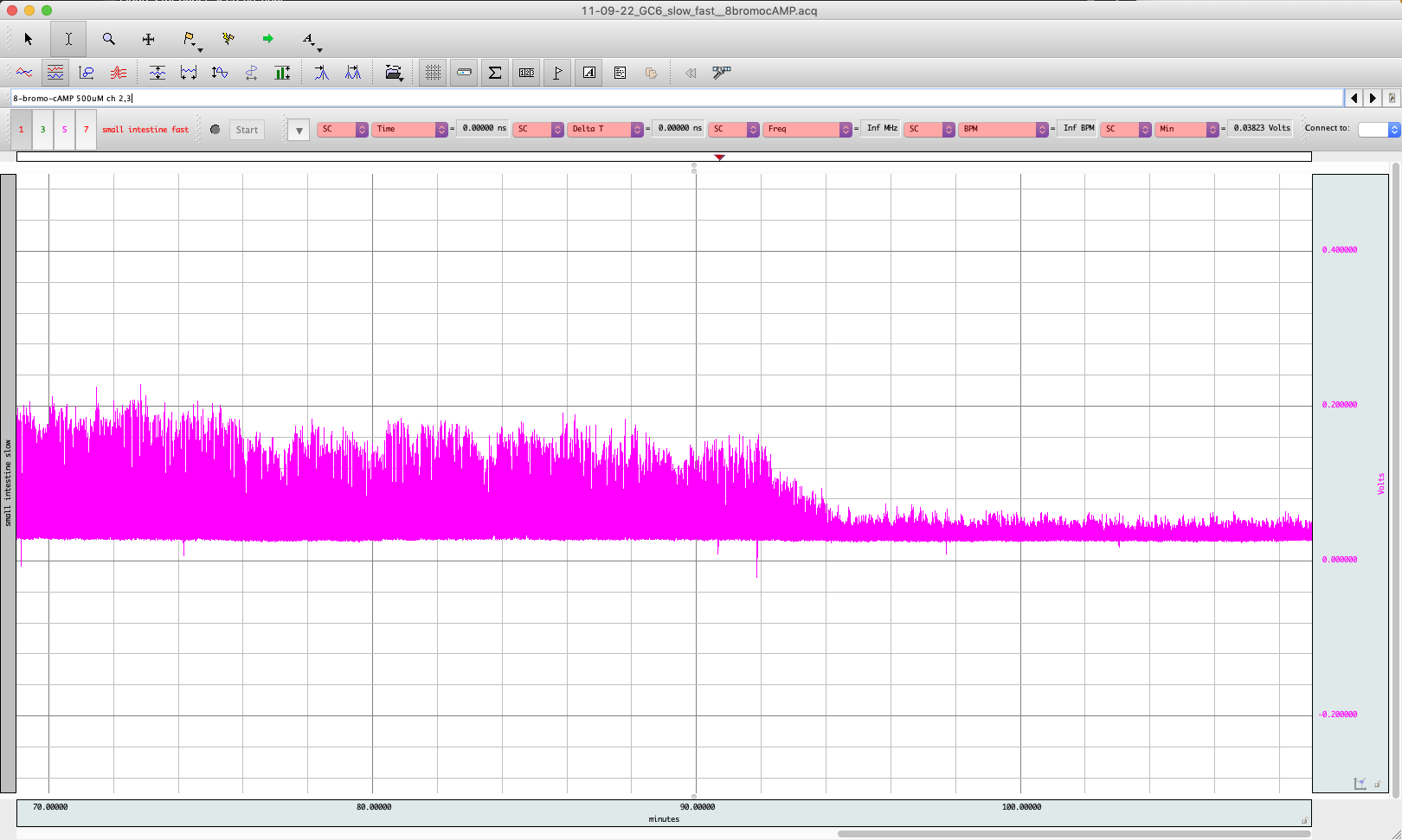

Supplement: Supplementary file 12 — Source data Fig. 9 [file 44319_2025_623_MOESM12_ESM.zip › Figure 9/9D-L/9G-I/traces_11-09-22__8bromocAMP.png]

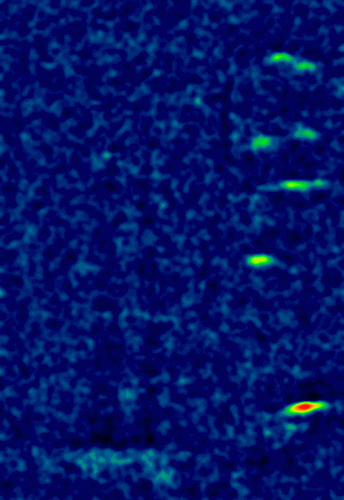

Supplement: Supplementary file 13 — Source data Fig. 10 [file 44319_2025_623_MOESM13_ESM.zip › Figure 10/10A-B/STMAPS_8bromocAMP_DMP/COLOR_2-6-25_40xGkitgc6_SI_IM_nicard300nm_ttx1uM_8bromo_500uM_3min_afterwash_STMap1-color.tif]

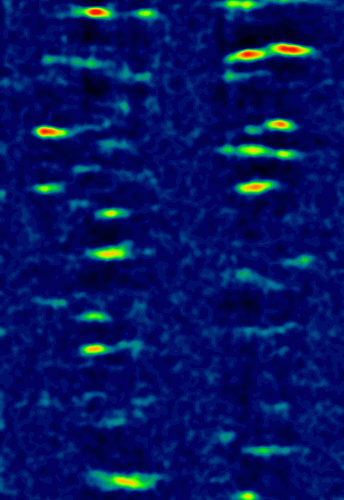

Supplement: Supplementary file 13 — Source data Fig. 10 [file 44319_2025_623_MOESM13_ESM.zip › Figure 10/10A-B/STMAPS_8bromocAMP_DMP/COLOR_2-6-25_40xGkitgc6_SI_IM_nicard300nm_ttx1uM_washoutCNTRL_STMap1-color.tif]

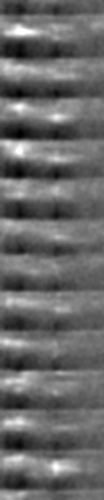

Supplement: Supplementary file 13 — Source data Fig. 10 [file 44319_2025_623_MOESM13_ESM.zip › Figure 10/10G-H/1-13-23_20x_gc6_SI_MY_8brcAMP500uM6min_STMap1.tif]

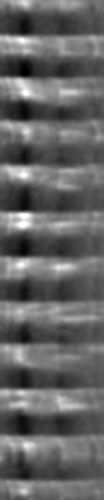

Supplement: Supplementary file 13 — Source data Fig. 10 [file 44319_2025_623_MOESM13_ESM.zip › Figure 10/10G-H/1-13-23_20x_gc6_SI_MY_STMap1.tif]

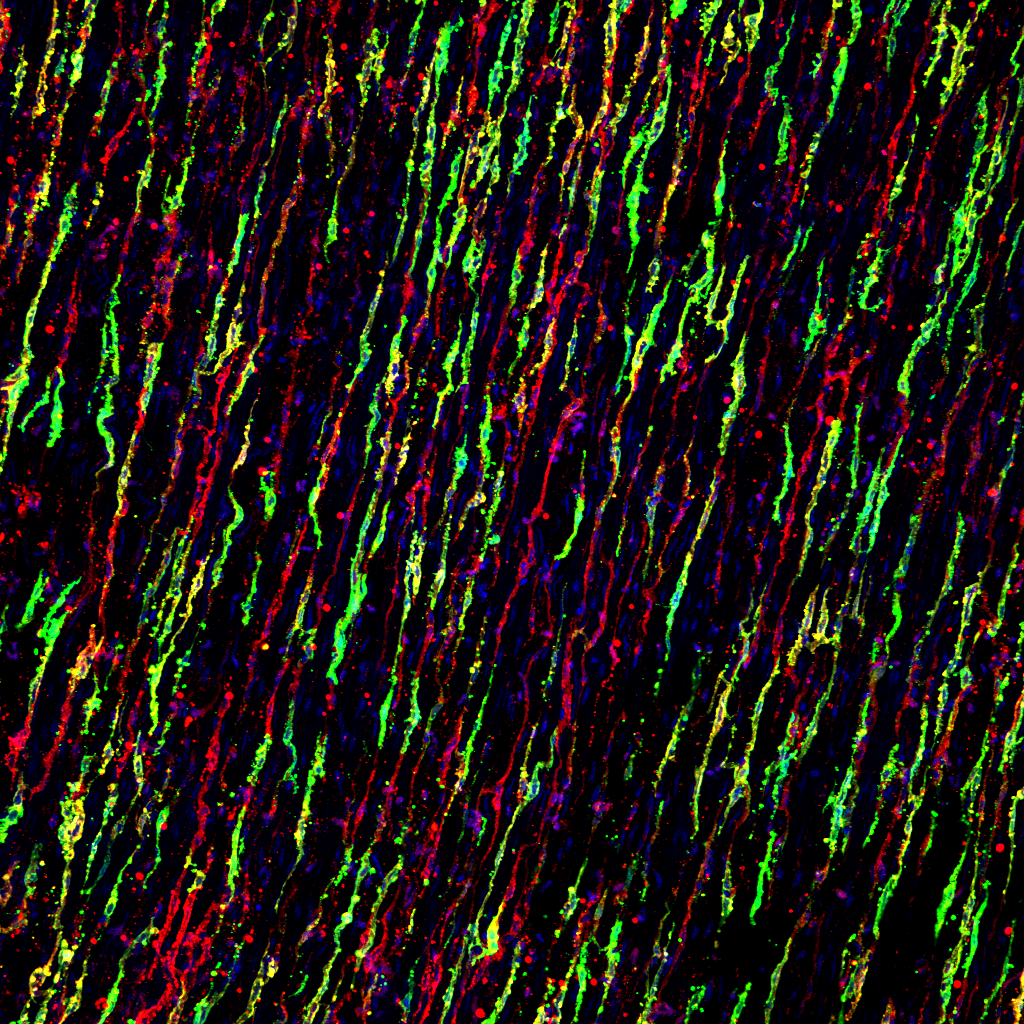

Supplement: Supplementary file 14 — Source data Fig. 11 [file 44319_2025_623_MOESM14_ESM.zip › Figure 11/11A-F/CAMPER-Kiticre/11-2-23_CAMPERkitSI_20xComposite (RGB).tif]

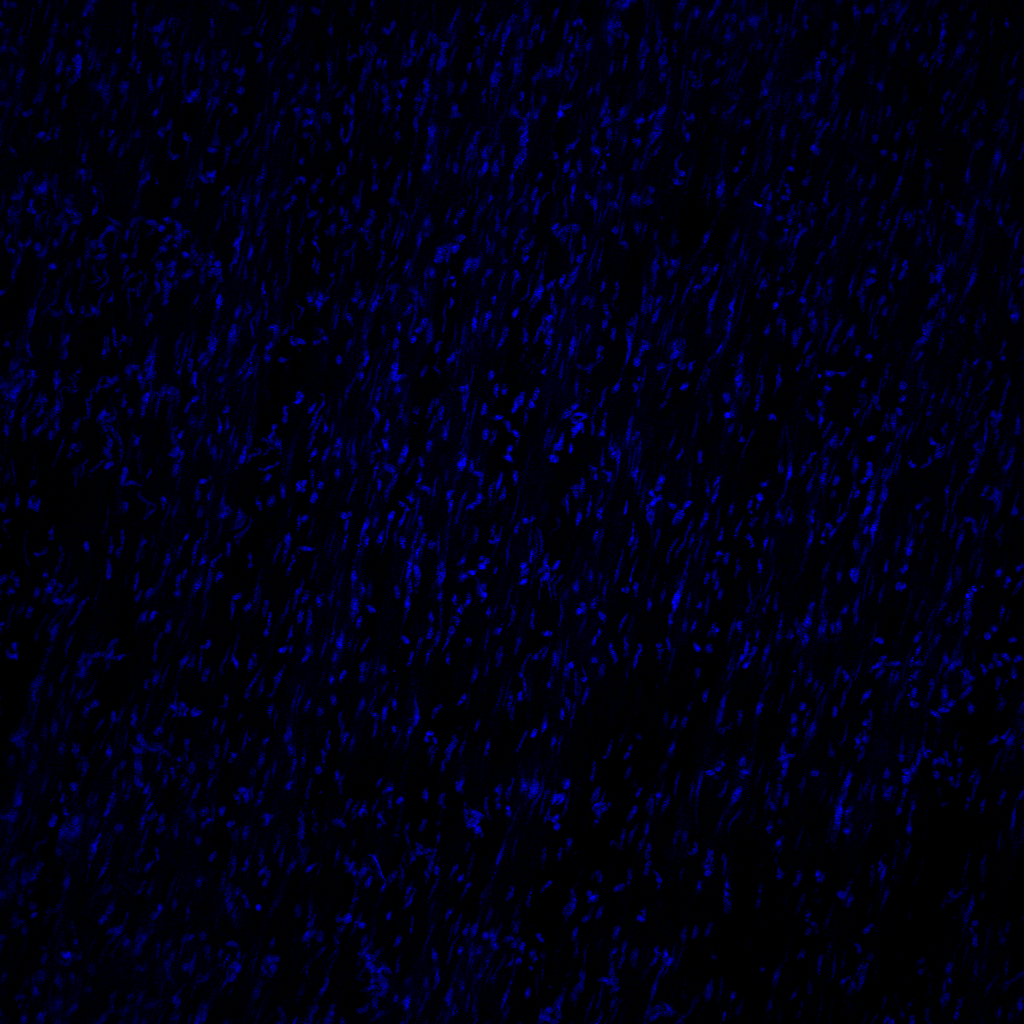

Supplement: Supplementary file 14 — Source data Fig. 11 [file 44319_2025_623_MOESM14_ESM.zip › Figure 11/11A-F/CAMPER-Kiticre/MAX_11-2-23CAMPERkitSIGFP488mscfr594_20x_IM002.nd2 - C=0.tif]

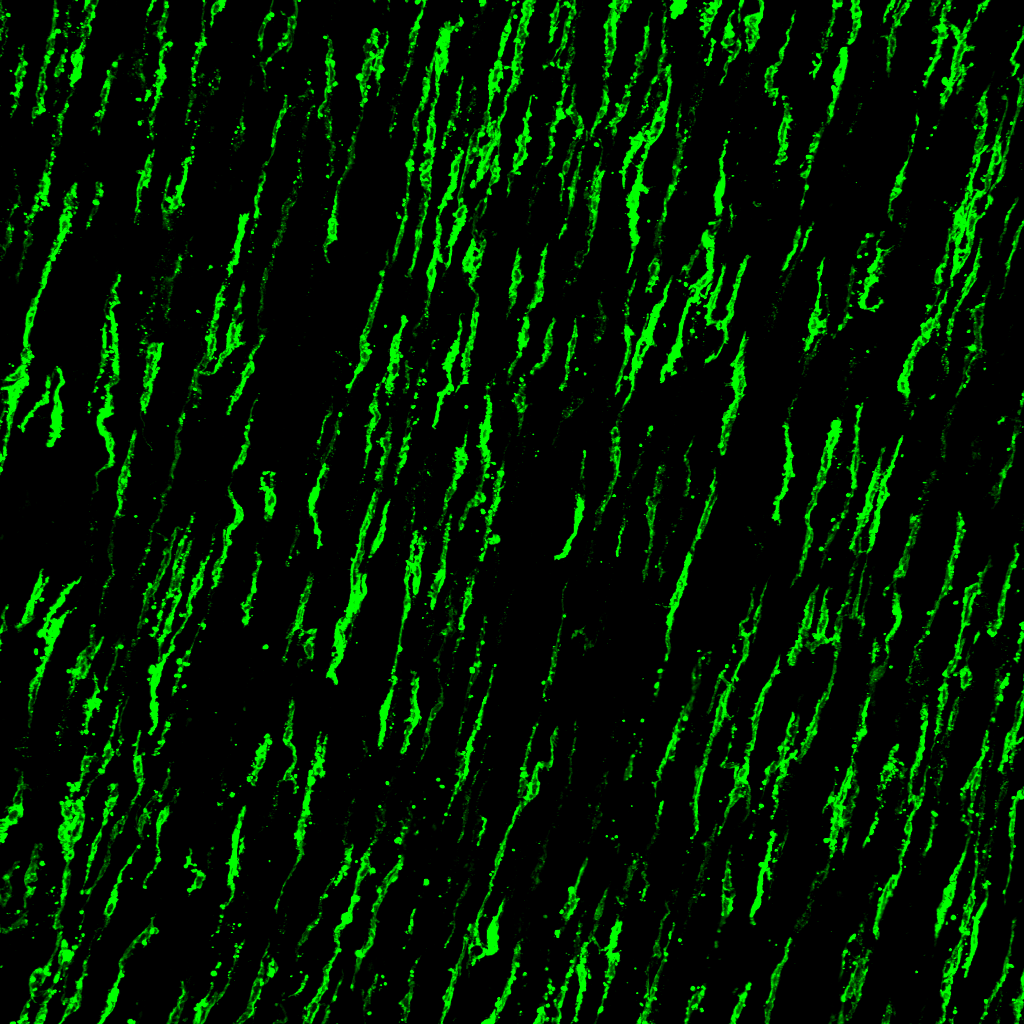

Supplement: Supplementary file 14 — Source data Fig. 11 [file 44319_2025_623_MOESM14_ESM.zip › Figure 11/11A-F/CAMPER-Kiticre/MAX_11-2-23CAMPERkitSIGFP488mscfr594_20x_IM002.nd2 - C=1.tif]

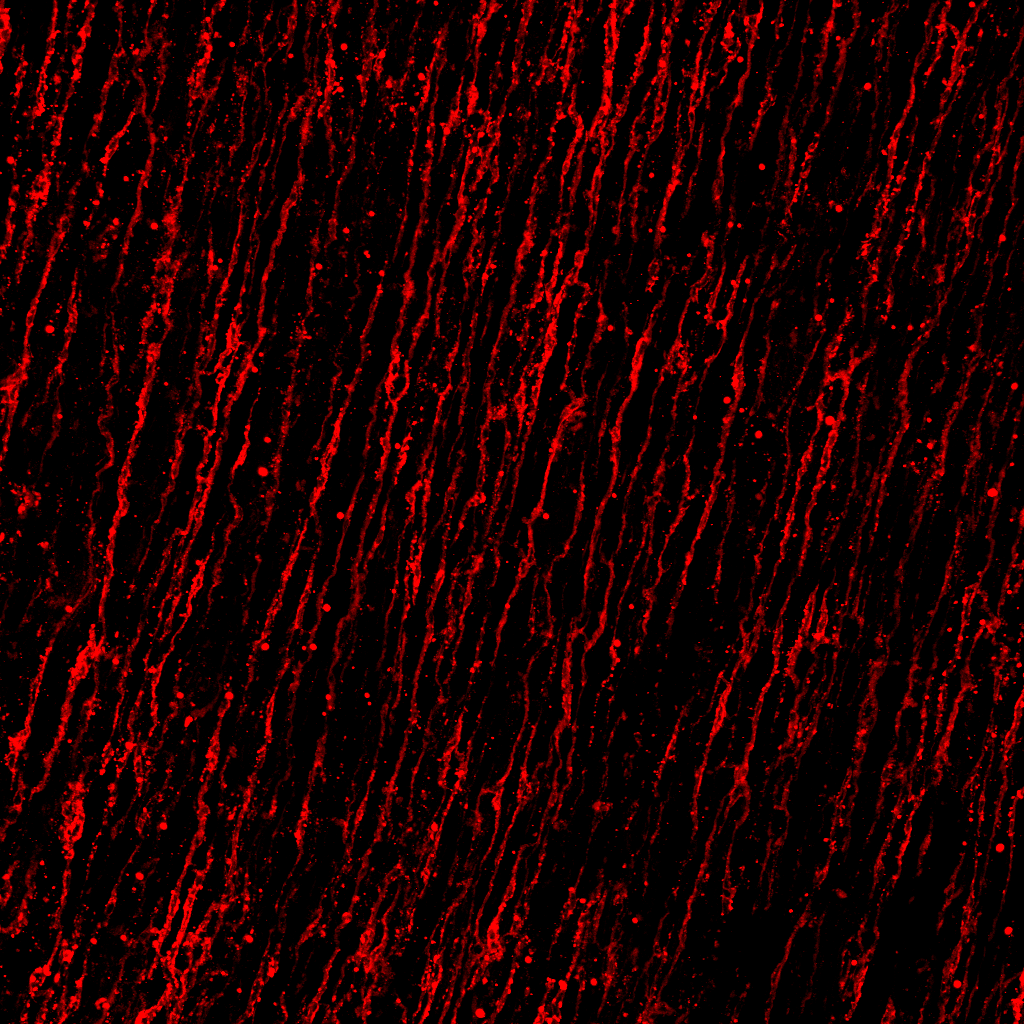

Supplement: Supplementary file 14 — Source data Fig. 11 [file 44319_2025_623_MOESM14_ESM.zip › Figure 11/11A-F/CAMPER-Kiticre/MAX_11-2-23CAMPERkitSIGFP488mscfr594_20x_IM002.nd2 - C=2.tif]

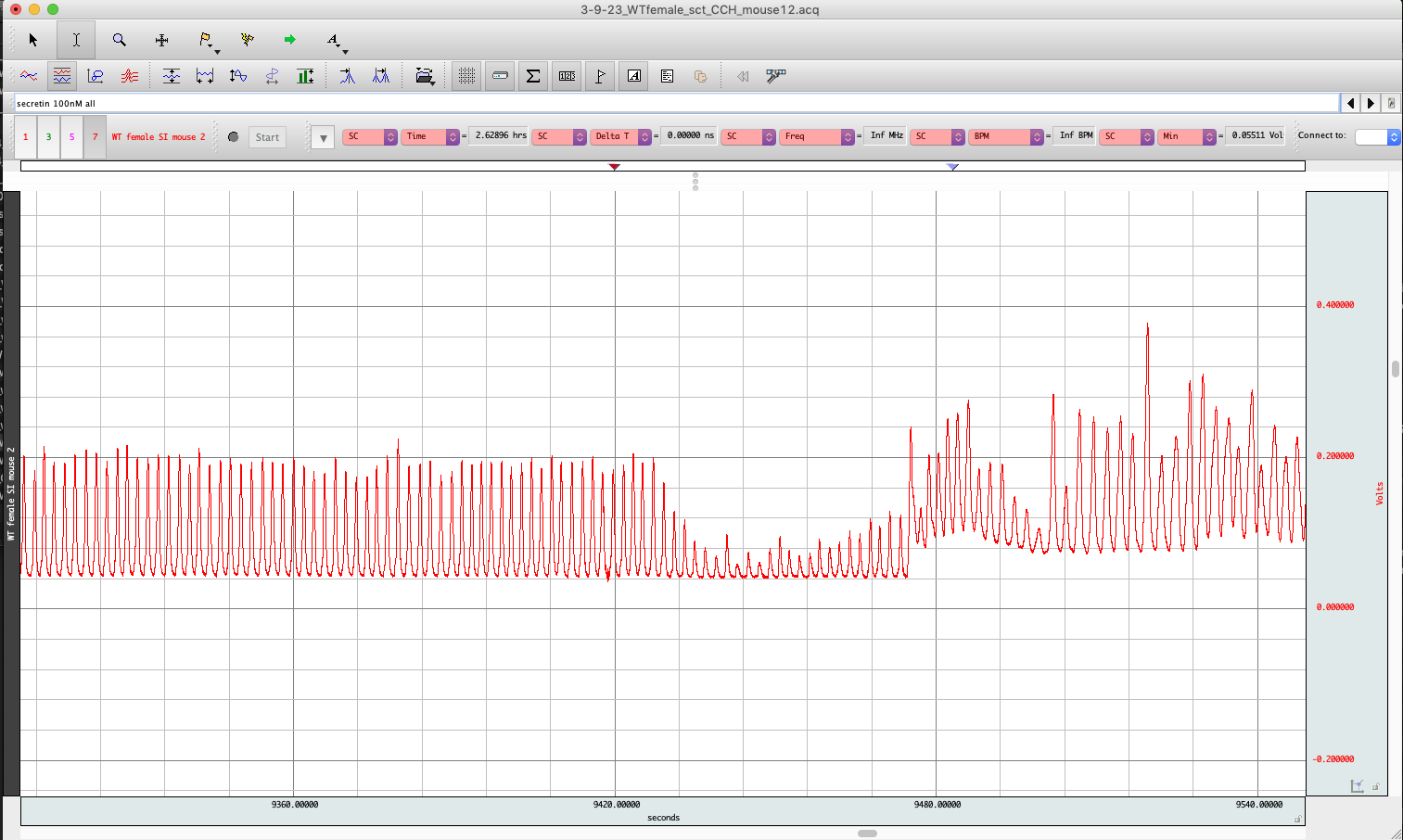

Supplement: Supplementary file 15 — Source data Fig. 12 [file 44319_2025_623_MOESM15_ESM.zip › Figure 12/12A-G/12A-B/trace_3-9-23_WTfemale_sct_control.png]

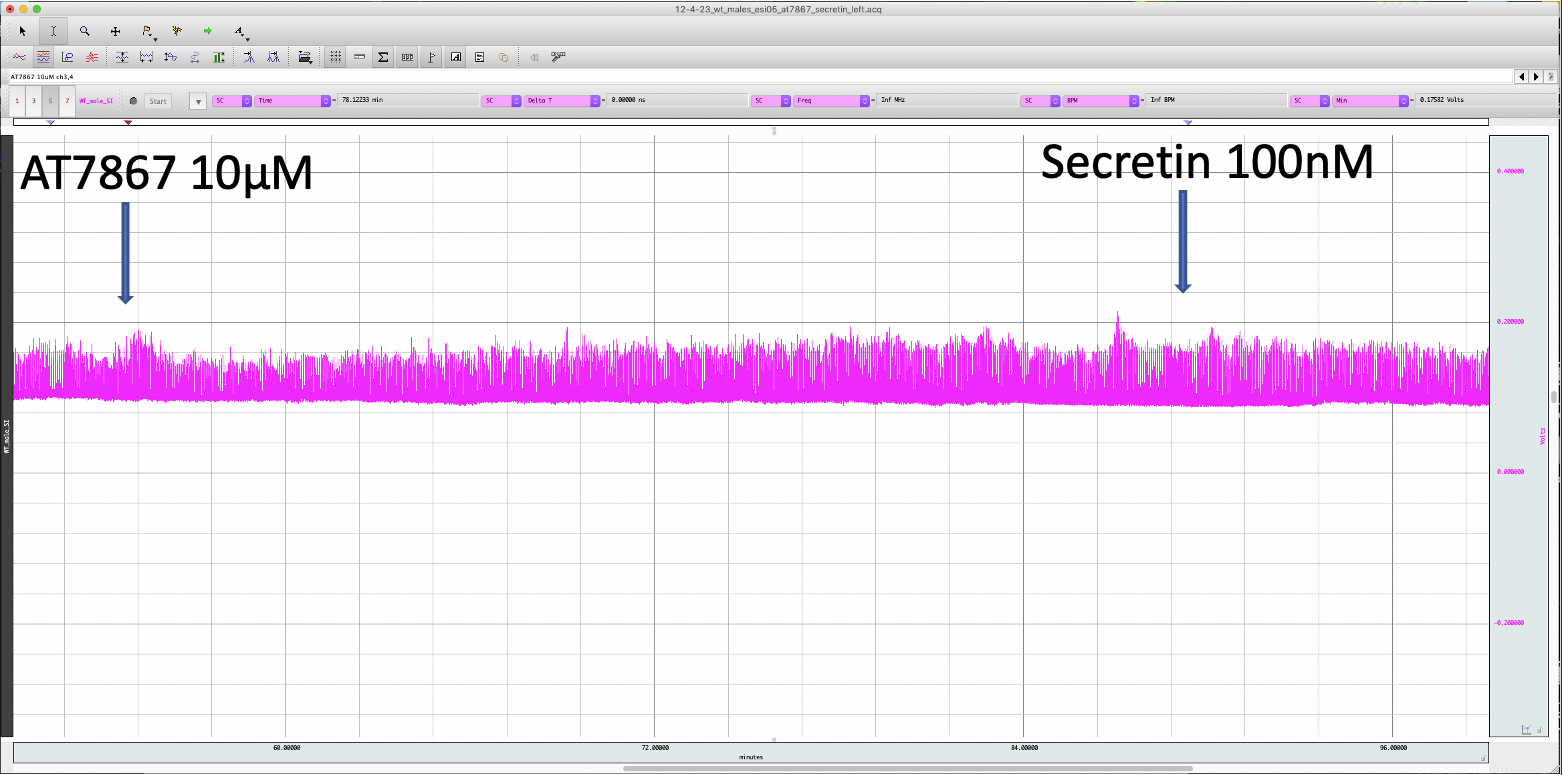

Supplement: Supplementary file 15 — Source data Fig. 12 [file 44319_2025_623_MOESM15_ESM.zip › Figure 12/12A-G/12C-D/Trace_12-4-23_wt_males_esi05_at7867_secretin_left.png]

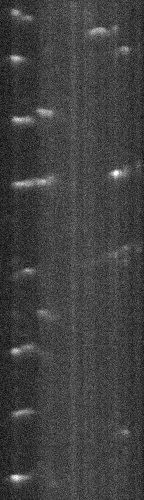

Supplement: Supplementary file 15 — Source data Fig. 12 [file 44319_2025_623_MOESM15_ESM.zip › Figure 12/12H-I/AT7867/8-22-23_GC6fkit_SI_MYIM_20x_AT786710uM30min_30sCNTRL_SCT100nMpuff_STMapIM1-after.tif]

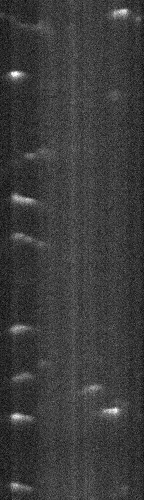

Supplement: Supplementary file 15 — Source data Fig. 12 [file 44319_2025_623_MOESM15_ESM.zip › Figure 12/12H-I/AT7867/8-22-23_GC6fkit_SI_MYIM_20x_AT786710uM30min_30sCNTRL_SCT100nMpuff_STMapIM1-before.tif]

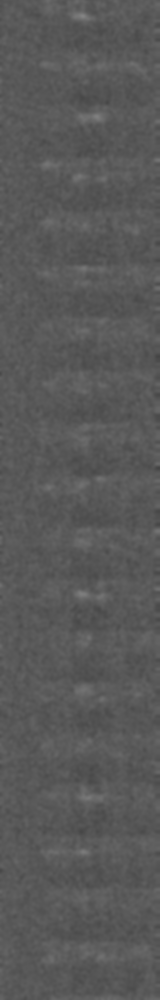

Supplement: Supplementary file 15 — Source data Fig. 12 [file 44319_2025_623_MOESM15_ESM.zip › Figure 12/12H-I/control/8-22-23_GC6fkit_SI_MYIM_20x_30sCNTRL_SCT100nMpuff_STMap2-after copy.tif]

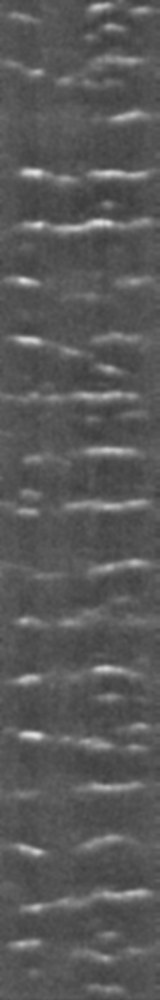

Supplement: Supplementary file 15 — Source data Fig. 12 [file 44319_2025_623_MOESM15_ESM.zip › Figure 12/12H-I/control/8-22-23_GC6fkit_SI_MYIM_20x_30sCNTRL_SCT100nMpuff_STMap2-before copy.tif]

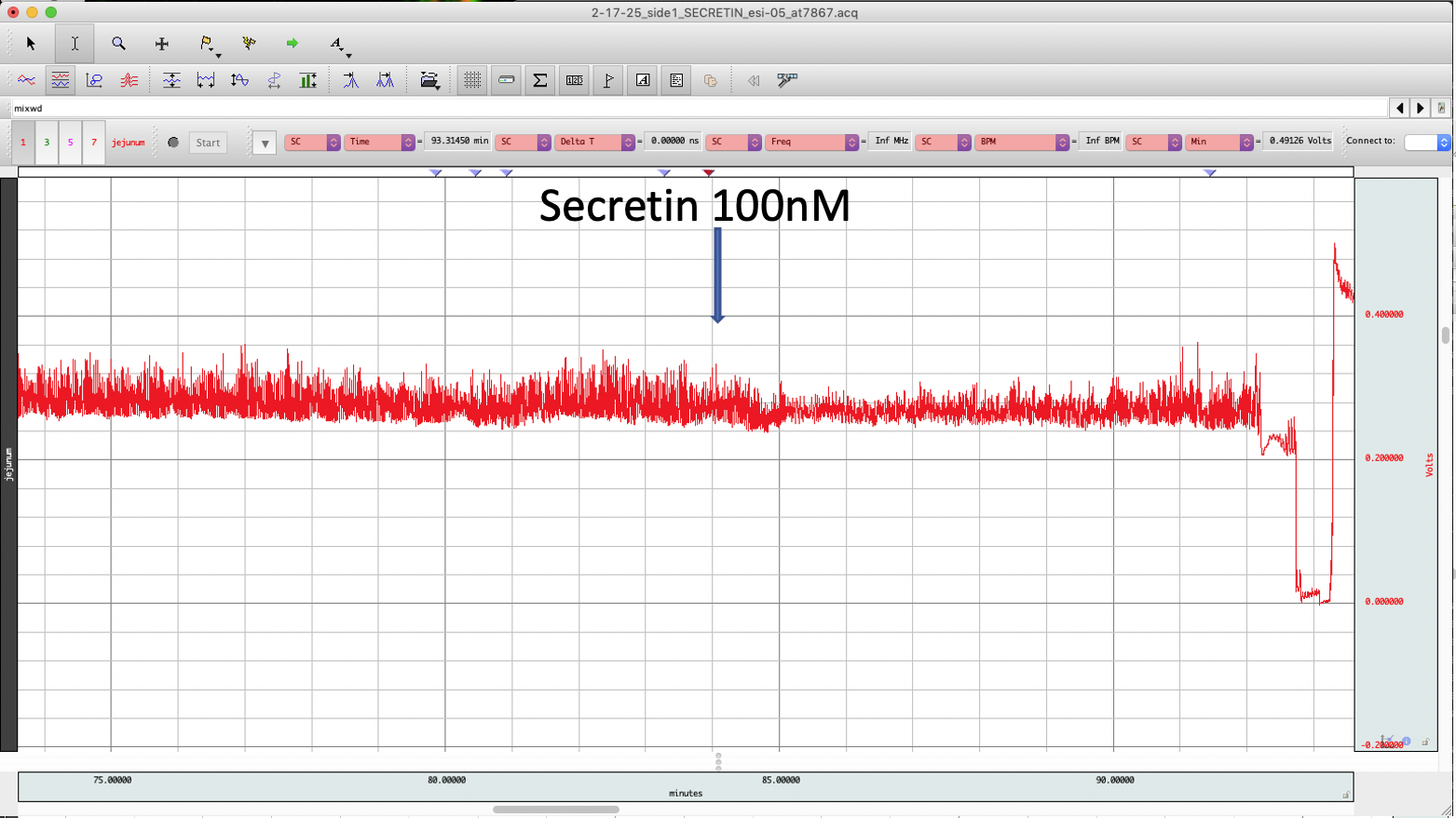

Supplement: Supplementary file 16 — Source data Fig. 13 [file 44319_2025_623_MOESM16_ESM.zip › Figure 13/13A-G/13A-B/controlTrace_2-17-25_side1_SECRETIN_esi-05_at7867.png]

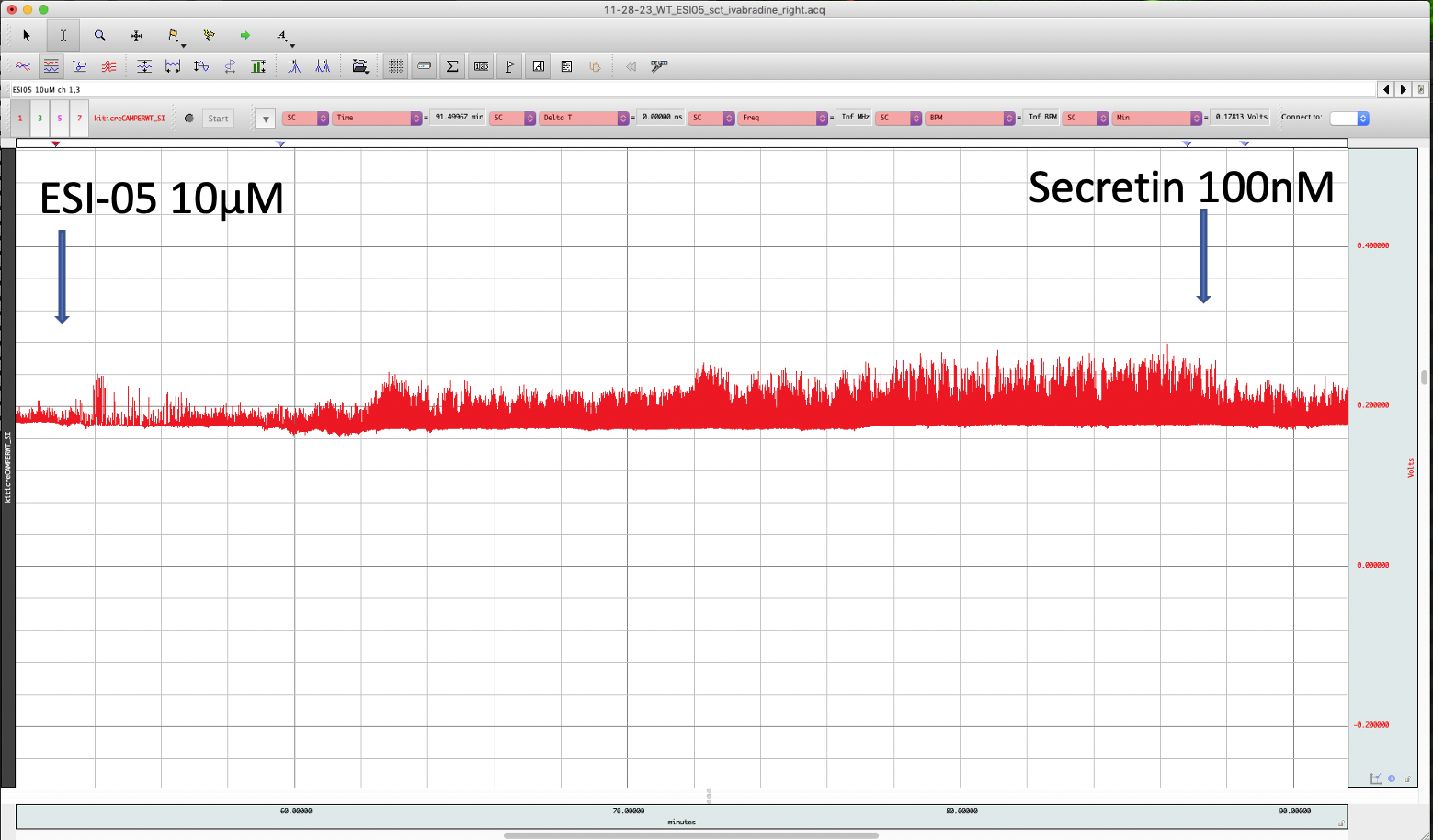

Supplement: Supplementary file 16 — Source data Fig. 13 [file 44319_2025_623_MOESM16_ESM.zip › Figure 13/13A-G/13C-D/trace_11-28-23_WT_ESI05_sct.png]

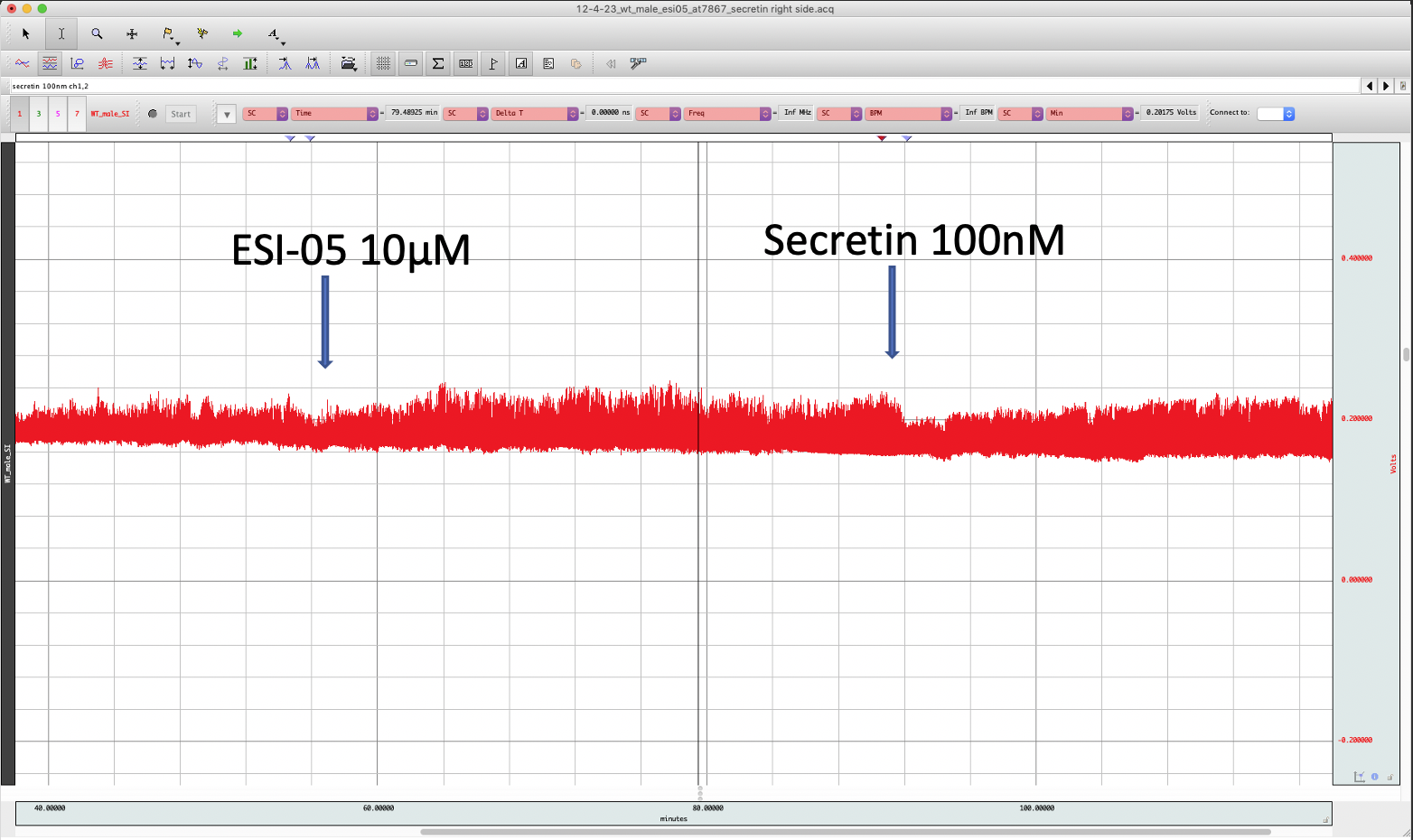

Supplement: Supplementary file 16 — Source data Fig. 13 [file 44319_2025_623_MOESM16_ESM.zip › Figure 13/13A-G/13C-D/trace_12-4-23_wt_male_esi05_at7867_secretin right side.png]

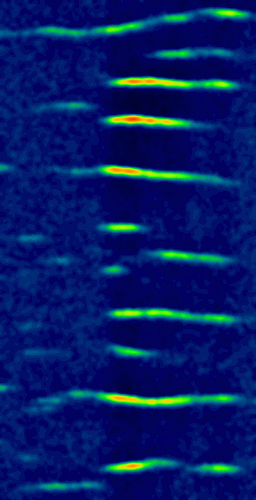

Supplement: Supplementary file 16 — Source data Fig. 13 [file 44319_2025_623_MOESM16_ESM.zip › Figure 13/13H-M/14H-I/COLOR_10-23-23_GC6KI_40x_IM_cntrl30s sct1umpuff_ESI05_10uM30min_STMapIM3-1 copy.tif]

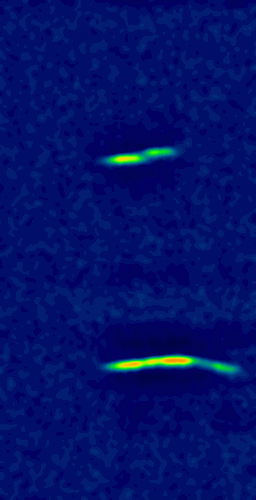

Supplement: Supplementary file 16 — Source data Fig. 13 [file 44319_2025_623_MOESM16_ESM.zip › Figure 13/13H-M/14H-I/COLOR_10-23-23_GC6KI_40x_IM_cntrl30s sct1umpuff_ESI05_10uM30min_STMapIM3-withSCT copy.tif]

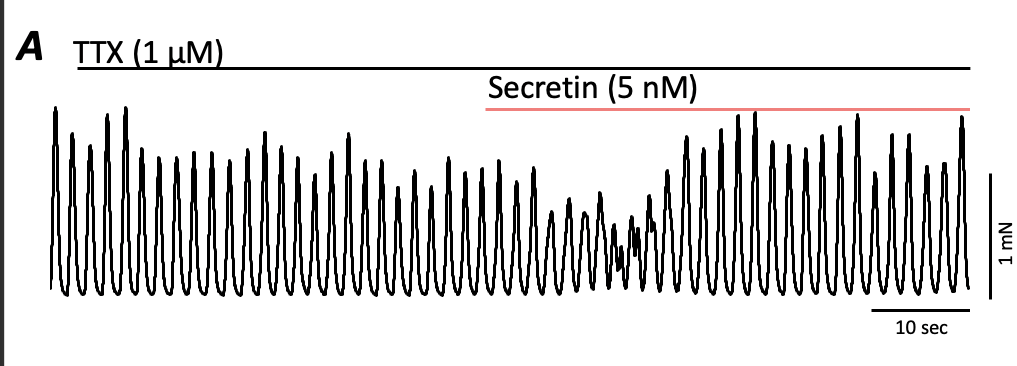

Supplement: Supplementary file 17 — EV and Appendix Figures Source Data [file 44319_2025_623_MOESM17_ESM.zip › EV_APPENDIX/APPENDIXS1/5nMTrace.png]

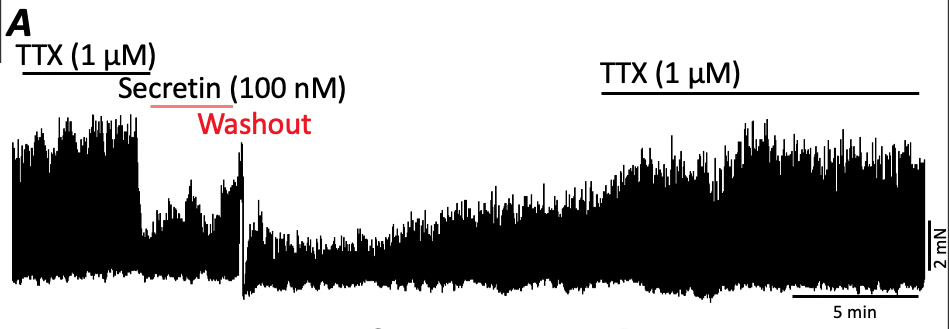

Supplement: Supplementary file 17 — EV and Appendix Figures Source Data [file 44319_2025_623_MOESM17_ESM.zip › EV_APPENDIX/APPENDIXS2/washouttrace.png]

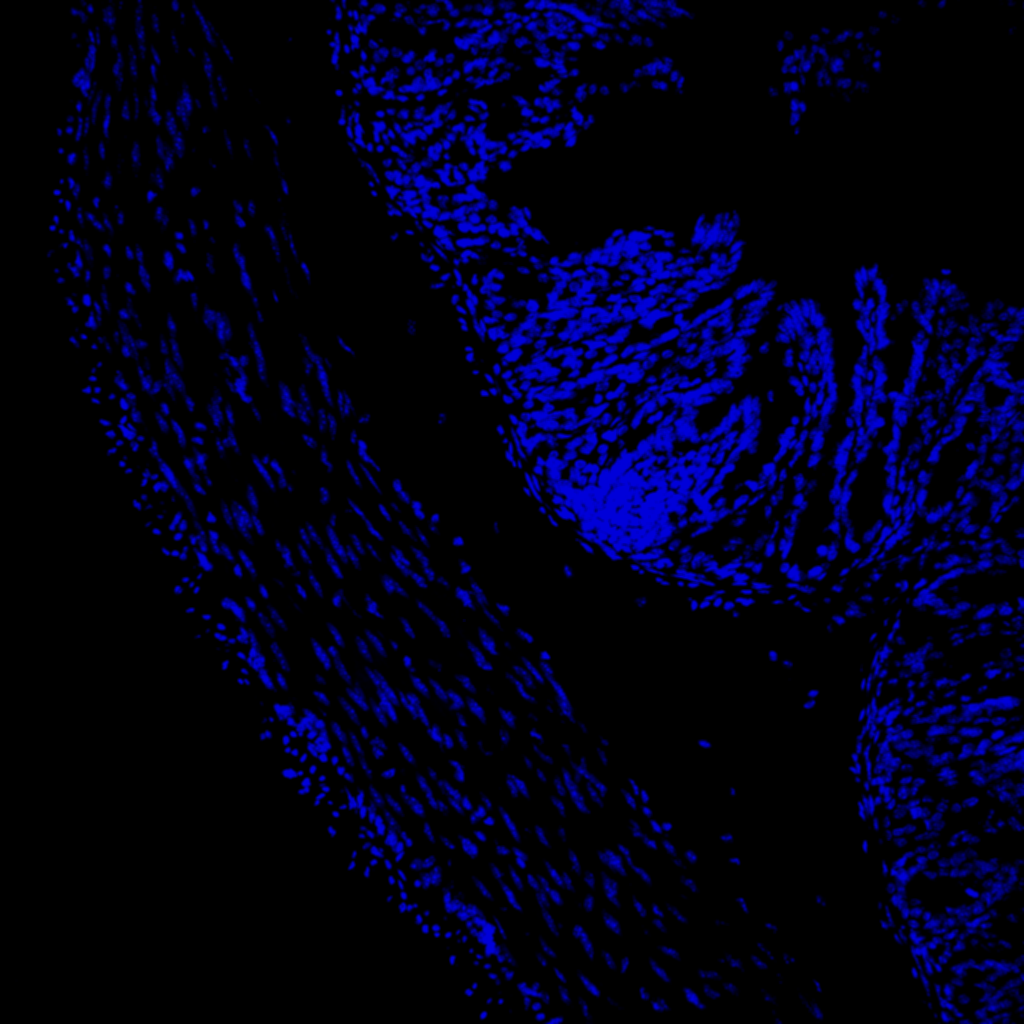

Supplement: Supplementary file 17 — EV and Appendix Figures Source Data [file 44319_2025_623_MOESM17_ESM.zip › EV_APPENDIX/APPENDIXS3/A-D/dapiMAX_5-1-23SI_RNASC_posCntrl_SI_cross_mscfr488_RNA594_20x.nd2 - C=0.tif]

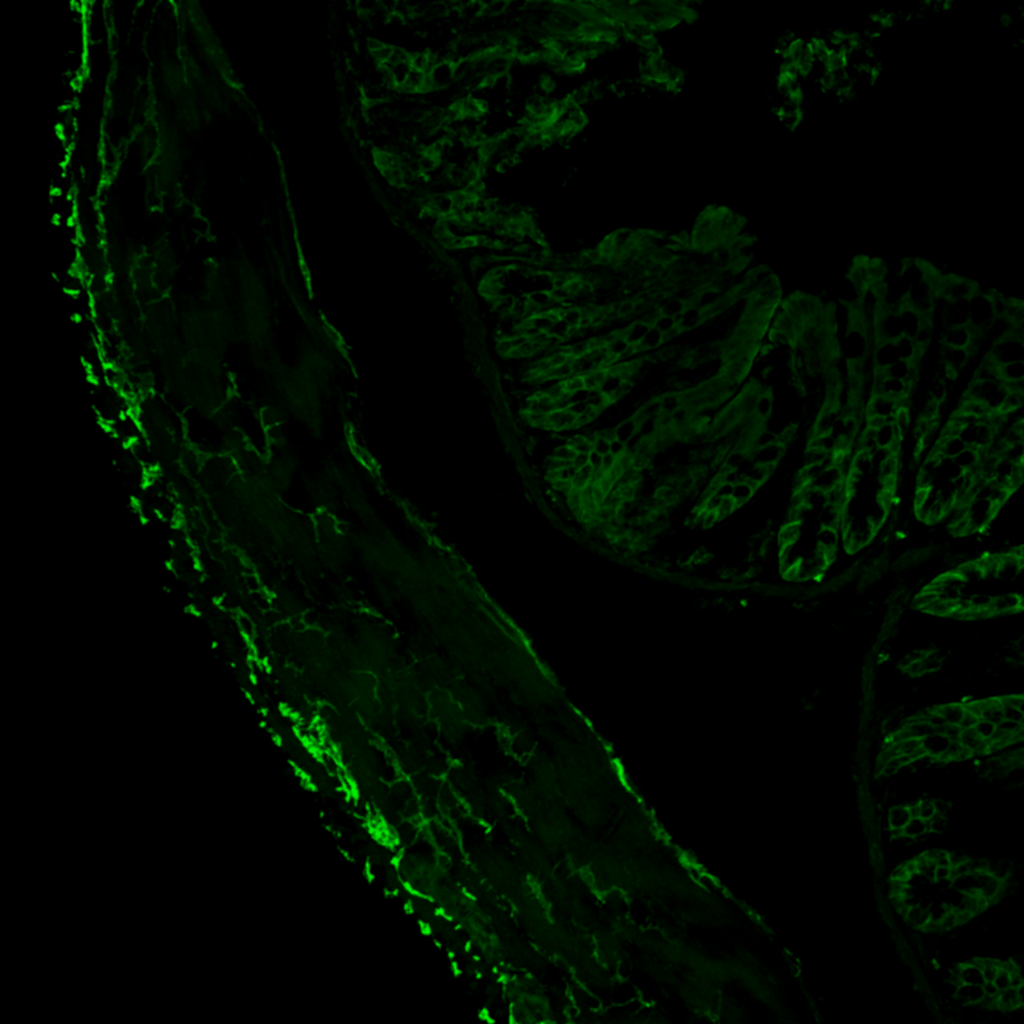

Supplement: Supplementary file 17 — EV and Appendix Figures Source Data [file 44319_2025_623_MOESM17_ESM.zip › EV_APPENDIX/APPENDIXS3/A-D/greenMAX_5-1-23SI_RNASC_posCntrl_SI_cross_mscfr488_RNA594_20x.nd2 - C=1.tif]

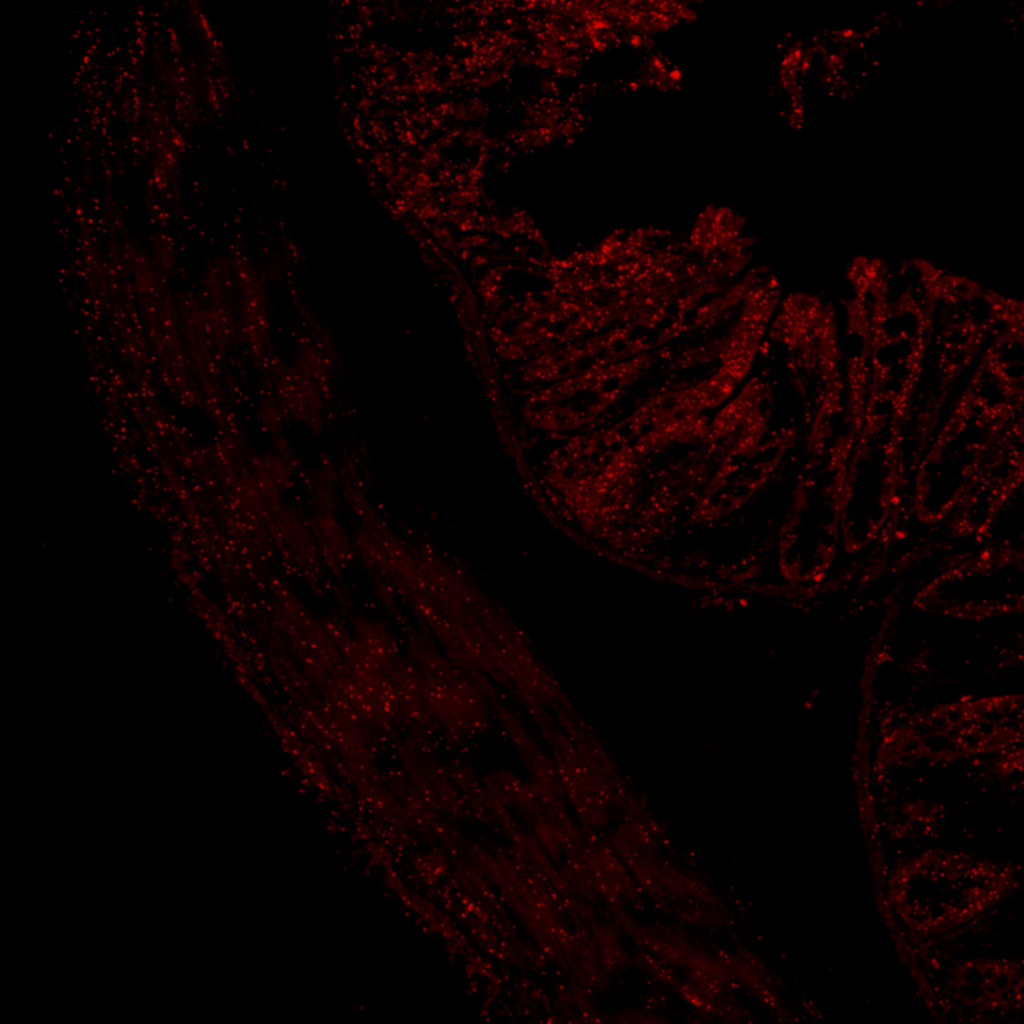

Supplement: Supplementary file 17 — EV and Appendix Figures Source Data [file 44319_2025_623_MOESM17_ESM.zip › EV_APPENDIX/APPENDIXS3/A-D/RED__MAX_5-1-23SI_RNASC_posCntrl_SI_cross_mscfr488_RNA594_20x.nd2 - C=2.tif]

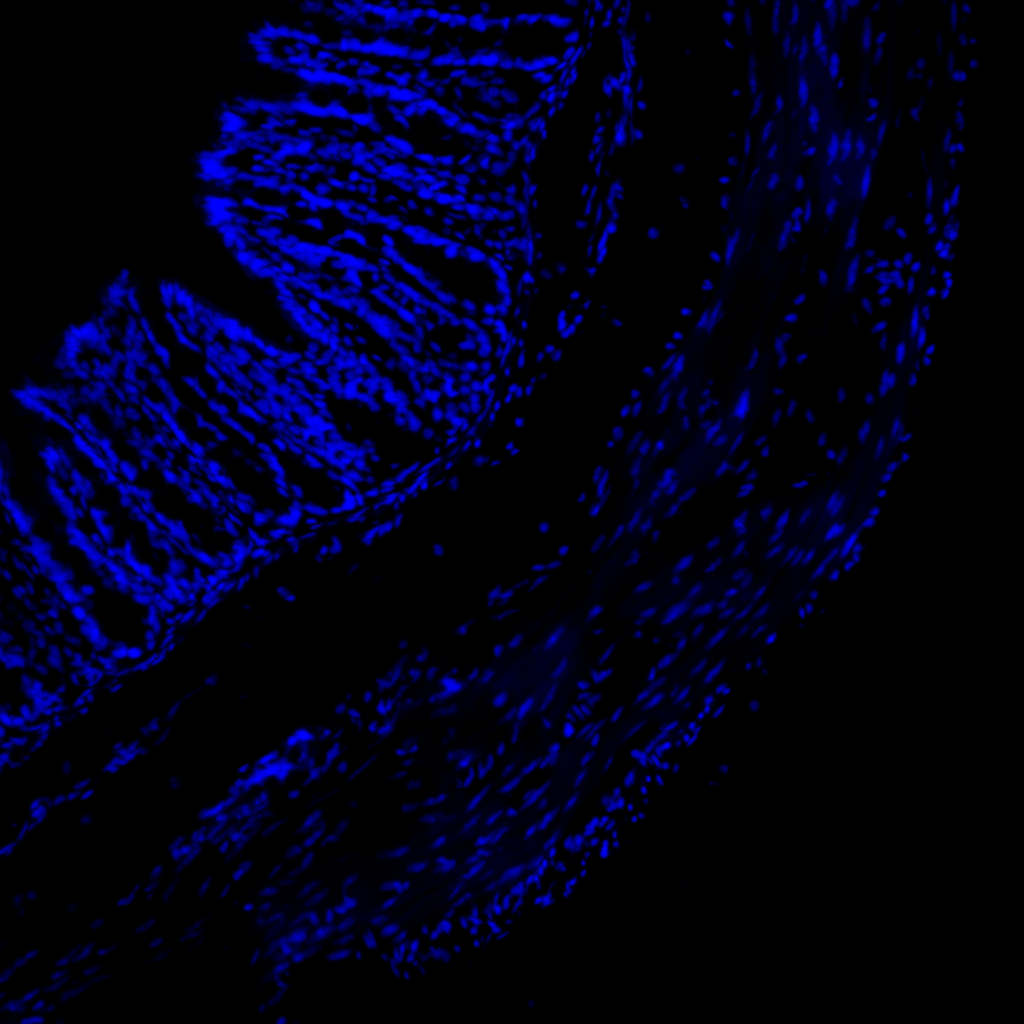

Supplement: Supplementary file 17 — EV and Appendix Figures Source Data [file 44319_2025_623_MOESM17_ESM.zip › EV_APPENDIX/APPENDIXS3/E-G/DAPI_AVG_5-1-23SI_RNASC_NegCntrl_SI_cross_mscfr488_RNA594_20x.nd2 - C=0.tif]

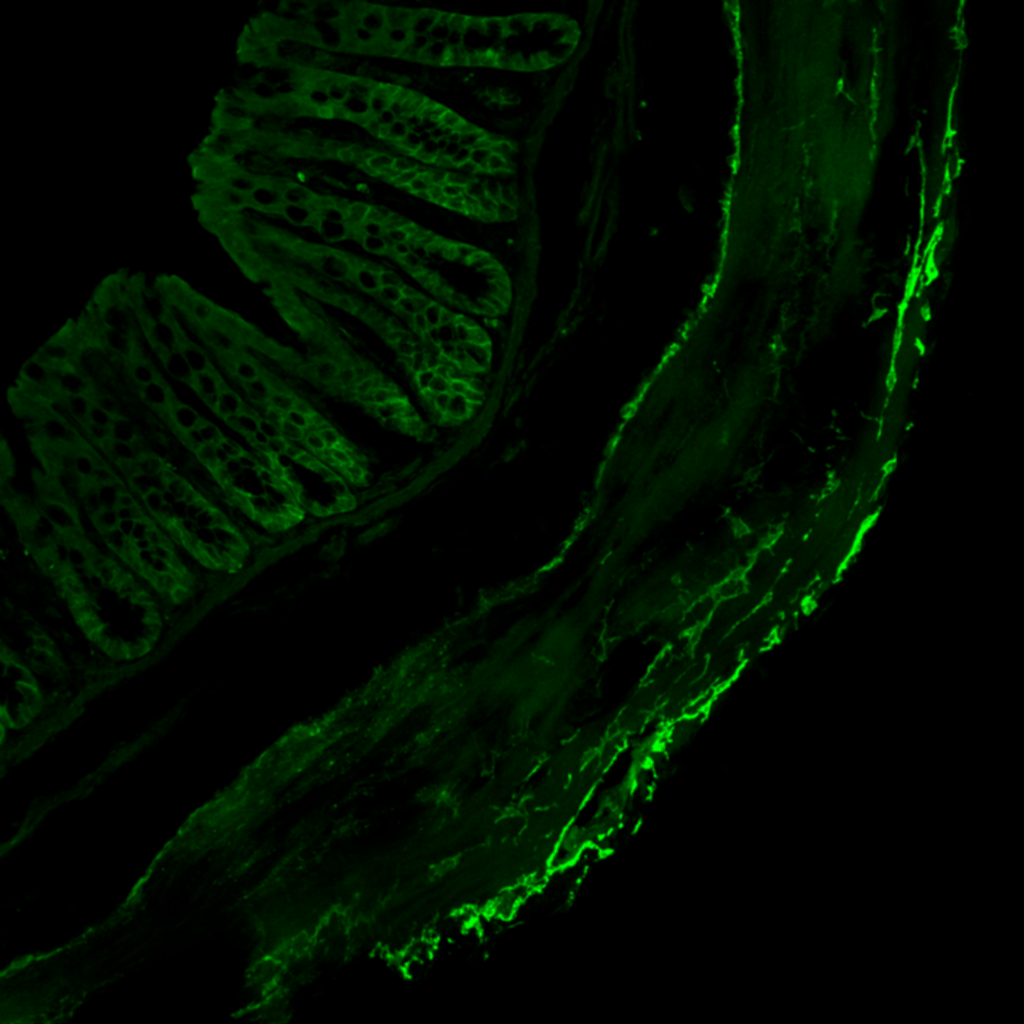

Supplement: Supplementary file 17 — EV and Appendix Figures Source Data [file 44319_2025_623_MOESM17_ESM.zip › EV_APPENDIX/APPENDIXS3/E-G/GREEN_MAX_5-1-23SI_RNASC_NegCntrl_SI_cross_mscfr488_RNA594_20x.nd2 - C=1.tif]

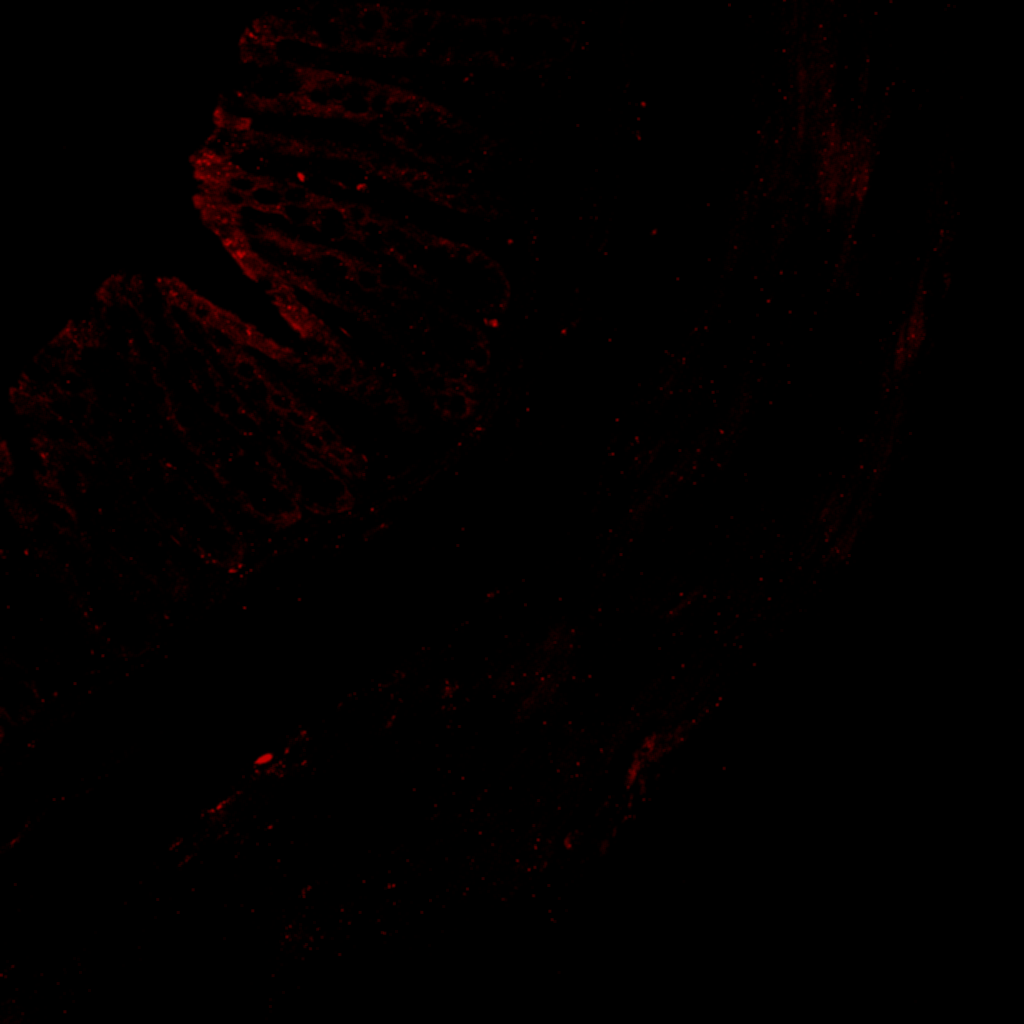

Supplement: Supplementary file 17 — EV and Appendix Figures Source Data [file 44319_2025_623_MOESM17_ESM.zip › EV_APPENDIX/APPENDIXS3/E-G/RED_MAX_5-1-23SI_RNASC_NegCntrl_SI_cross_mscfr488_RNA594_20x.nd2 - C=2.tif]

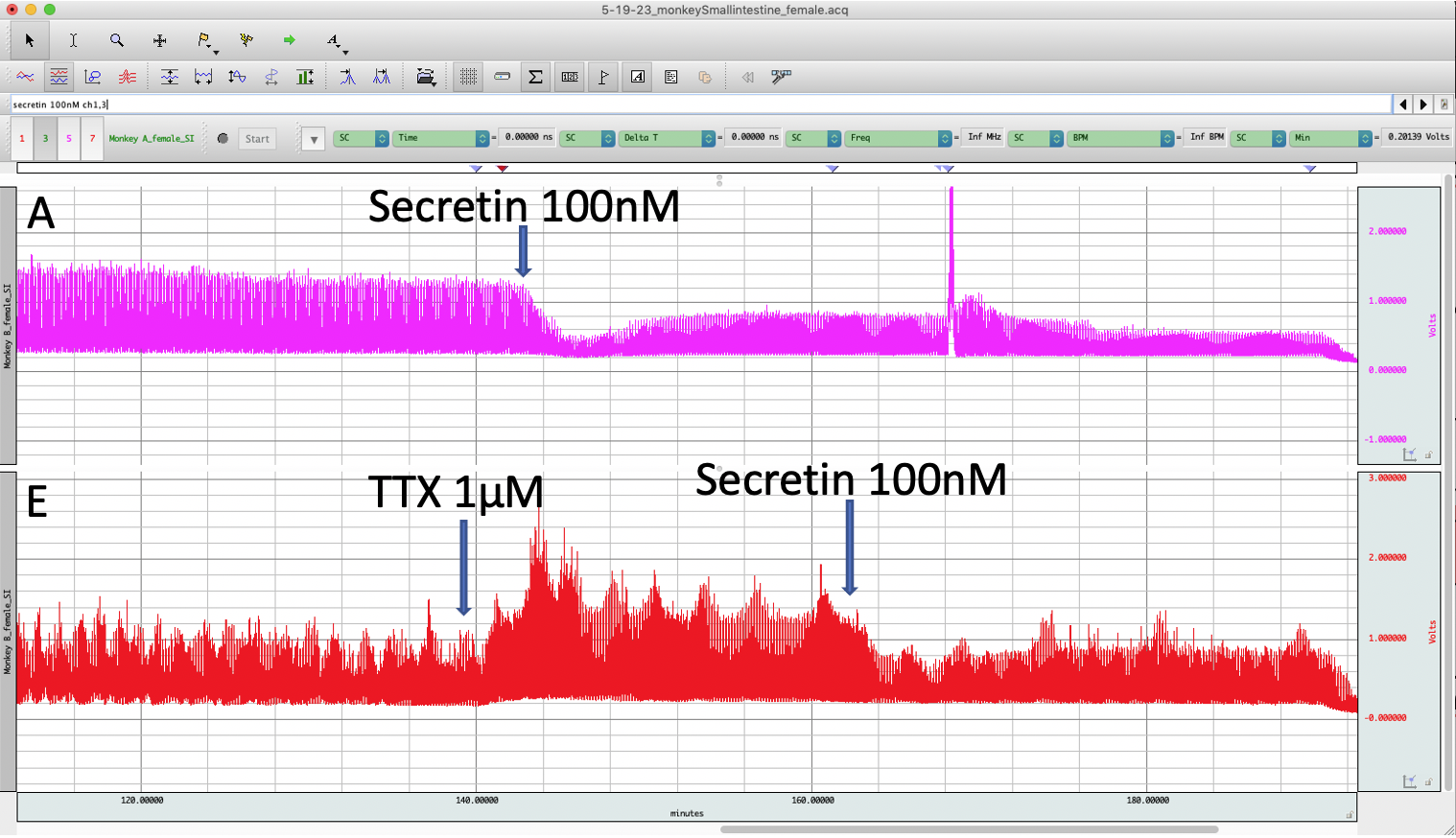

Supplement: Supplementary file 17 — EV and Appendix Figures Source Data [file 44319_2025_623_MOESM17_ESM.zip › EV_APPENDIX/EV1/EV1A-H/traces_5-19-23_monkeySmallintestine_female.png]

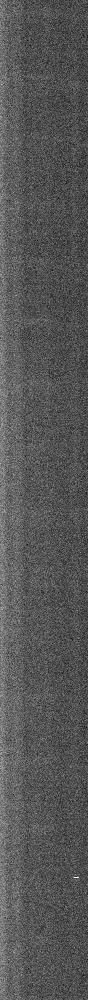

Supplement: Supplementary file 17 — EV and Appendix Figures Source Data [file 44319_2025_623_MOESM17_ESM.zip › EV_APPENDIX/EV2/EV2A-B/11-6-23_40x_gc6_IMMY_nicard200nM_sctpuff_35scntrl__STMapIM-AFTER copy.tif]

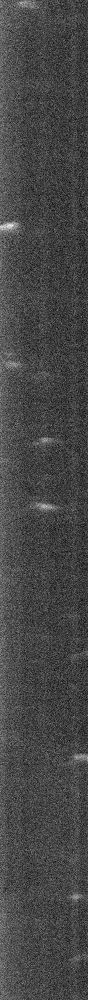

Supplement: Supplementary file 17 — EV and Appendix Figures Source Data [file 44319_2025_623_MOESM17_ESM.zip › EV_APPENDIX/EV2/EV2A-B/11-6-23_40x_gc6_IMMY_nicard200nM_sctpuff_35scntrl__STMapIM-BEFORE copy.tif]

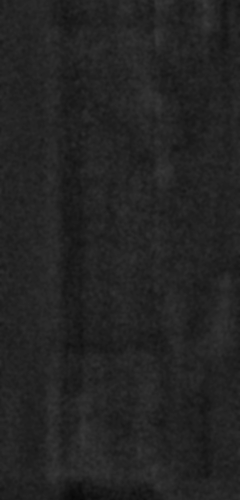

Supplement: Supplementary file 17 — EV and Appendix Figures Source Data [file 44319_2025_623_MOESM17_ESM.zip › EV_APPENDIX/EV3/EV3C-D/11-14-23_40xT2_im_gc6_Nic200nMcntrl35s_sct100nM_thapsigargin25min_STMapIM1-after.tif]

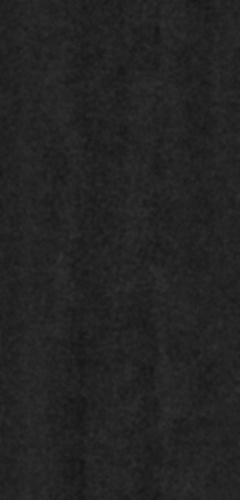

Supplement: Supplementary file 17 — EV and Appendix Figures Source Data [file 44319_2025_623_MOESM17_ESM.zip › EV_APPENDIX/EV3/EV3C-D/11-14-23_40xT2_im_gc6_Nic200nMcntrl35s_sct100nM_thapsigargin25min_STMapIM1-before.tif]
