# Supplementary material for: Secretin targets interstitial cells of Cajal to regulate intestinal contractions
Source: EMBO Rep. 2025 Nov 6;26(23):6015–43. doi: 10.1038/s44319-025-00623-1 (PMC12678811; doi:10.1038/s44319-025-00623-1)
Supplement: Supplementary file 18 — Expanded View Figures [file 44319_2025_623_MOESM18_ESM.pdf]

# Expanded View Figures

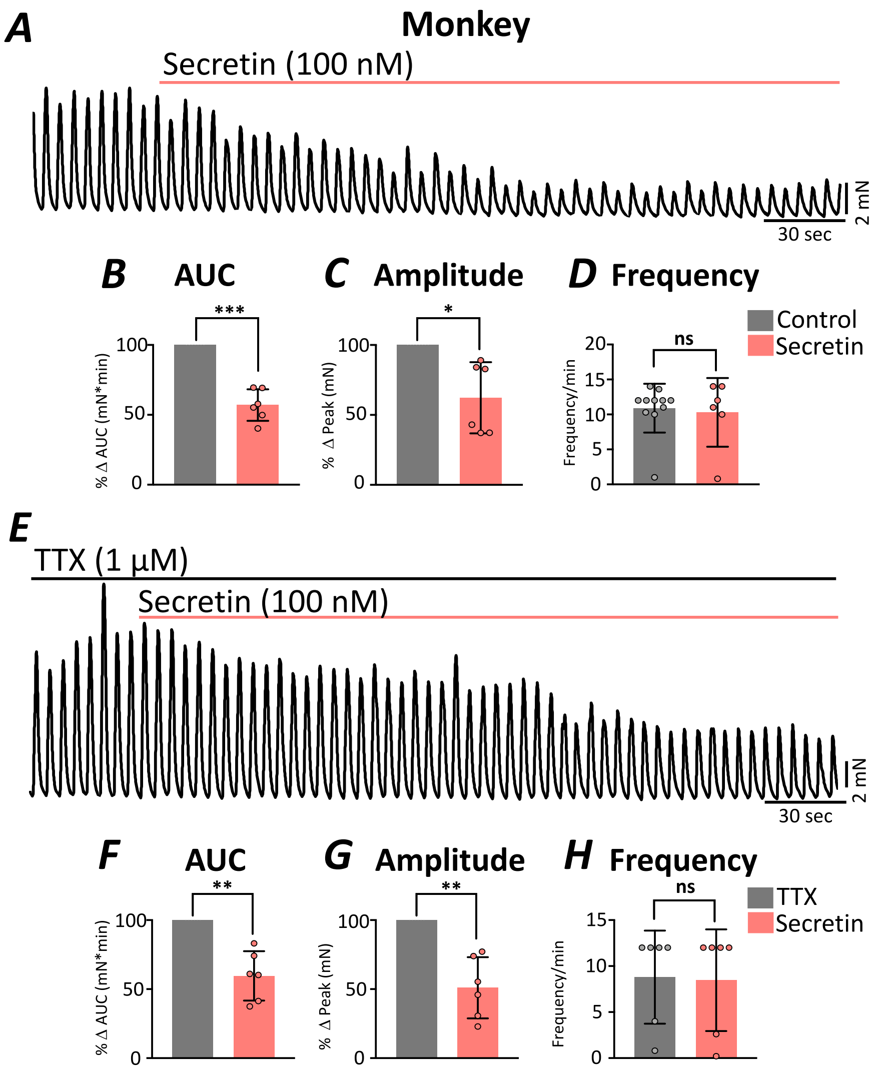


**Figure EV1. Secretin inhibits contractions of small intestinal muscles from Cynomolgus monkeys.**

(**A**) Contractions of monkey jejunal muscles were reduced by secretin (100 nM). (**B**) The area under the curve (AUC; mN*min) and (**C**) amplitude (mN) of contractions were reduced, however, (**D**) the frequency ($min^{-1}$) was unaffected. (**E**) The response to secretin was unaffected by TTX (1µM). (**F**) AUC and (**G**) amplitude of contractions were reduced but (**H**) the frequency of contractions was unaffected. All data were normalized to controls except contraction frequency. The data are plotted as mean ± SEM, and significance was determined using paired *t* test. ***$P = 0.0002$ (**B**); *$P = 0.0150$ (**C**); **$P = 0.0026$ (**F**); **$P = 0.0029$ (**G**); Strips = 7, $n = 3$ for control and strips = 6, $n = 4$ for TTX preparations. Source data are available online for this figure.

© The Author(s)

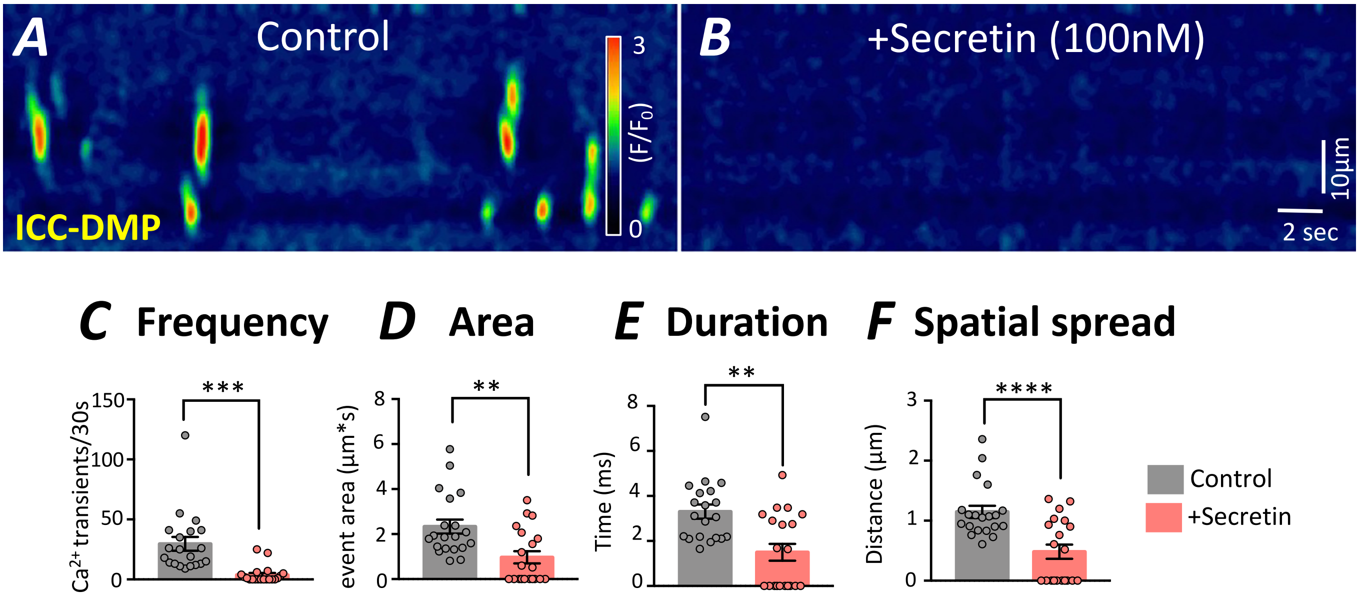


**Figure EV2. Secretin inhibits $Ca^{2+}$ transients in ICC-DMP.**

$Ca^{2+}$ transients of ICC-DMP were recorded from Kit-iCre-GCaMP6f mouse small intestinal muscles. (**A**) ICC-DMP GCaMP6f signals plotted as STMaps and (**B**) after the addition of secretin (100 nM). Secretin inhibited $Ca^{2+}$ transients: (**C**) frequency of $Ca^{2+}$ transients (per 30 s), (**D**) area of events (µm*s), (**E**) duration of events (ms), and (**F**) spatial spread of $Ca^{2+}$ transients (µm). The data are plotted as mean ± SEM, and significance was determined using paired *t* test. ***$P = 0.0002$ (**C**); **$P = 0.0015$ (**D**); **$P = 0.0026$ (**E**); ****$P < 0.0001$ (**F**); cells = 20, $n = 10$. Source data are available online for this figure.

 © The Author(s)

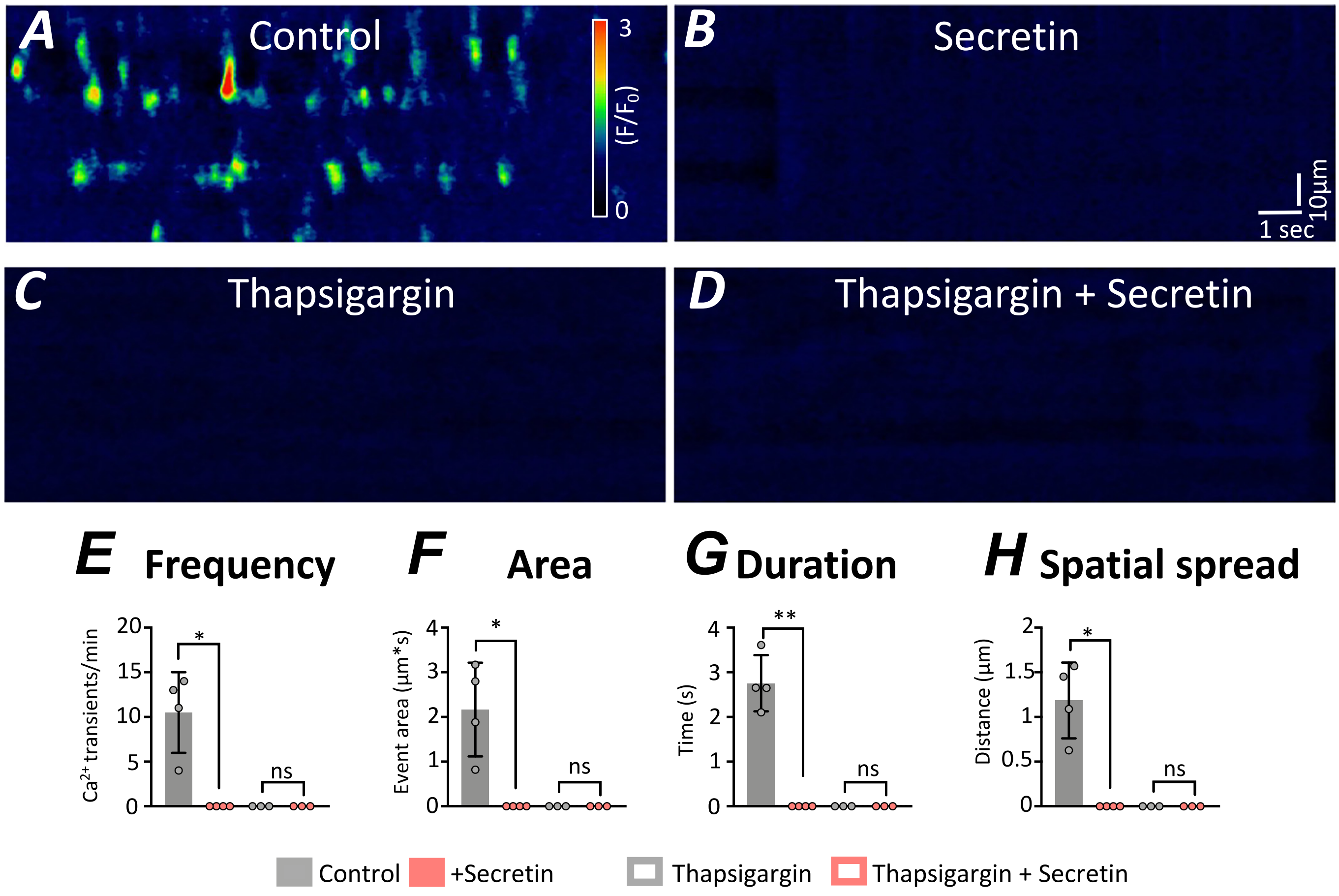


**Figure EV3. Secretin acts on $Ca^{2+}$ release mechanisms in ICC-DMP.**

$Ca^{2+}$ transients of ICC-DMP were recorded from Kit-iCre-GCaMP6f mouse small intestinal muscles. (**A**) ICC-DMP GCaMP6f signals plotted as STMaps and (**B**) after the addition of secretin (100 nM). (**C**) Thapsigargin (10 μM) inhibited $Ca^{2+}$ transients in ICC-DMP, and no further effect was noted after addition of secretin (100 nM) (**D**). All transients were inhibited: (**E**) frequency of $Ca^{2+}$ transients (per 30 s), (**F**) area of events (μm*s), (**G**) duration of events (ms), and (**H**) spatial spread of $Ca^{2+}$ transients (μm). The data are plotted as mean ± SEM, and significance was determined using paired *t* test. *=*P* = *0.0187* (**E**); **P* = 0.0257 (**F**); ***P* = 0.0031 (**G**); **P* = 0.0113(**H**); for controls, cells = 4, *n* = 4 for thapsigargin, cells = 3, *n* = 3. Source data are available online for this figure.

© The Author(s)
